# Mapping the unicellular transcriptome of the ascending thoracic aorta to changes in mechanosensing and mechanoadaptation during aging

**DOI:** 10.1111/acel.14197

**Published:** 2024-06-02

**Authors:** Cristobal F. Rivera, Yasmeen M. Farra, Michele Silvestro, Steven Medvedovsky, Jacqueline Matz, Muhammad Yogi Pratama, John Vlahos, Bhama Ramkhelawon, Chiara Bellini

**Affiliations:** ^1^ Department of Surgery, Division of Vascular and Endovascular Surgery New York University Langone Medical Center New York New York USA; ^2^ Department of Cell Biology New York University Langone Medical Center New York New York USA; ^3^ Department of Bioengineering Northeastern University Boston Massachusetts USA

**Keywords:** collagen remodeling, distensibility, elastic energy, inflammation, Piezo‐1, tissue stiffness, vasoconstriction

## Abstract

Aortic stiffening is an inevitable manifestation of chronological aging, yet the mechano‐molecular programs that orchestrate region‐ and layer‐specific adaptations along the length and through the wall of the aorta are incompletely defined. Here, we show that the decline in passive cyclic distensibility is more pronounced in the ascending thoracic aorta (ATA) compared to distal segments of the aorta and that collagen content increases in both the medial and adventitial compartments of the ATA during aging. The single‐cell RNA sequencing of aged ATA tissues reveals altered cellular senescence, remodeling, and inflammatory responses accompanied by enrichment of T‐lymphocytes and rarefaction of vascular smooth muscle cells, compared to young samples. T lymphocyte clusters accumulate in the adventitia, while the activation of mechanosensitive Piezo‐1 enhances vasoconstriction and contributes to the overall functional decline of ATA tissues. These results portray the immuno‐mechanical aging of the ATA as a process that culminates in a stiffer conduit permissive to the accrual of multi‐gerogenic signals priming to disease development.

## Graphical abstract

The decline in passive cyclic distensibility of the aorta during aging is more pronounced in the ascending thoracic aorta (ATA) compared to distal segments. Aging induces cellular senescence, remodeling, and inflammatory responses accompanied by enrichment of adventitial T‐lymphocytes and rarefaction of medial vascular smooth muscle cells in ATA tissues. Activation of mechanosensitive Piezo‐1 enhances vasoconstriction and contributes to the overall functional decline of the aged ATA.

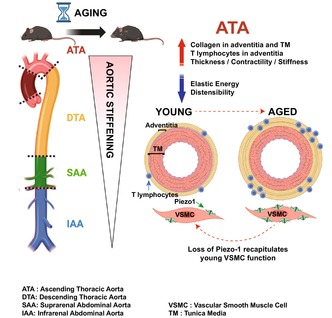

**Abbreviations**
ATA: ascending thoracic aorta
BSA: bovine serum albumin
CVD: cardiovascular disease
DAPI: 4',6‐diamidino‐2‐phenylindole
DTA: descending thoracic aorta
EC: endothelial cell
ECM: extracellular matrix
FITC: fluorescein isothiocyanate
F‐l: force versus length
FSC‐A: forward scatter area
FSC‐H: forward scatter height
GEM: gel bead in emulsion
HBSS: Hanks buffered salt solution
IAA: infrarenal abdominal aorta
IACUC: institutional animal care and use committee
IPA: ingenuity pathway analysis
MTC: Masson's trichrome
MYH11: myosin heavy chain 11
NIH: national institute of health
OCT: optimal cutting temperature
PBS: phosphate buffer solution
P‐d: pressure versus diameter
PFA: paraformaldehyde
PWV: pulse wave velocity
SAA: suprarenal abdominal aorta
scRNA‐seq: single‐cell RNA sequencing
SEM: standard error of the mean
UMAP: uniform manifold approximation and projection
UMI: unique molecular identifier
VSMC: vascular smooth muscle cell
VVG: Verhoeff Van Gieson

## 1 INTRODUCTION

Epidemiological evidence has established that cardiovascular disease (CVD) is a leading cause of death amongst adults 65 and older (Jaul & Barron, 2017) and advanced age is one of the dominant risk factors that fuels the development of CVD (Rodgers et al., 2019). Concurrent clinical findings of elevated body mass index, hypertension, hyperlipidemia, and smoking further enhance the risk of acute cardiovascular events in the aging population, including myocardial infarction, ischemic stroke, and heart failure (Lind et al., 2018). Considering that chronological aging cannot be reversed, dissection of age‐responsive molecular and cellular pathways and their impact on the function of central arteries is crucial to mitigate the damaging effects of aging and intercept aortic disease via spatio‐temporal pharmacological interventions.

Vascular function is essentially mechanical in nature, and mechanical metrics have emerged as reliable estimators of cardiovascular age. Amongst those, structural stiffening of elastic arteries is an independent predictor of all‐causes and cardiovascular mortality in hypertensive patients, and it is a risk factor for CVD in both hypertensive and normotensive populations (Boutouyrie et al., 2008; Laurent et al., 2006; Sutton‐Tyrrell et al., 2005). A spatially‐averaged measure of structural stiffness, pulse wave velocity (PWV), doubles between 20 and 80 years of age (O'Rourke & Hashimoto, 2007), thereby elevating the workload on the heart and contributing to negative downstream effects on microvasculature and organs (Ferruzzi et al., 2018). Enzymatic degradation, fragmentation, and calcification of medial elastic material (Schlatmann & Becker, 1977), deposition of collagen throughout the aortic wall (Fleenor et al., 2012; Greenberg, 1986; Schlatmann & Becker, 1977), and cross‐linking of structural proteins due to non‐enzymatic glycation (Schleicher et al., 1997; Sims et al., 1996) synergistically contribute to the age‐related structural stiffening of conduit arteries. Notwithstanding these insights, the broad spectrum of molecular and cellular adaptations that accompany a compromised aged vasculature has yet to be fully resolved (Rammos et al., 2014).

We determined that the structural stiffening of the mouse aorta occurs progressively with aging and heterogeneously along the artery, with the proximal ascending thoracic aorta (ATA) experiencing the largest distensibility decline. Single‐cell RNA sequencing of ATA samples isolated from 12‐ and 84‐week‐old mice revealed that signaling pathways such as cellular senescence, inflammation, vascular remodeling, endothelial dysfunction, and T lymphocyte responses are overrepresented in aged mice. Furthermore, Piezo‐1 activation enhances the vasoconstrictive response of medial vascular smooth muscle cells (VSMC) and partly mediates the age‐induced functional decline of ATA tissues. These processes collectively culminate in a stiffer conduit with reduced ability to store elastic energy for diastolic blood flow augmentation. Our work thus provides direct evidence of intrinsic vascular maladaptations during aging and identifies Piezo‐1 as one of the gerogenic culprits of pathological extracellular matrix (ECM) remodeling.

## 2 RESULTS

### 2.1 Accelerated structural stiffening in the ascending portion of the aged aorta

**FIGURE 1:**
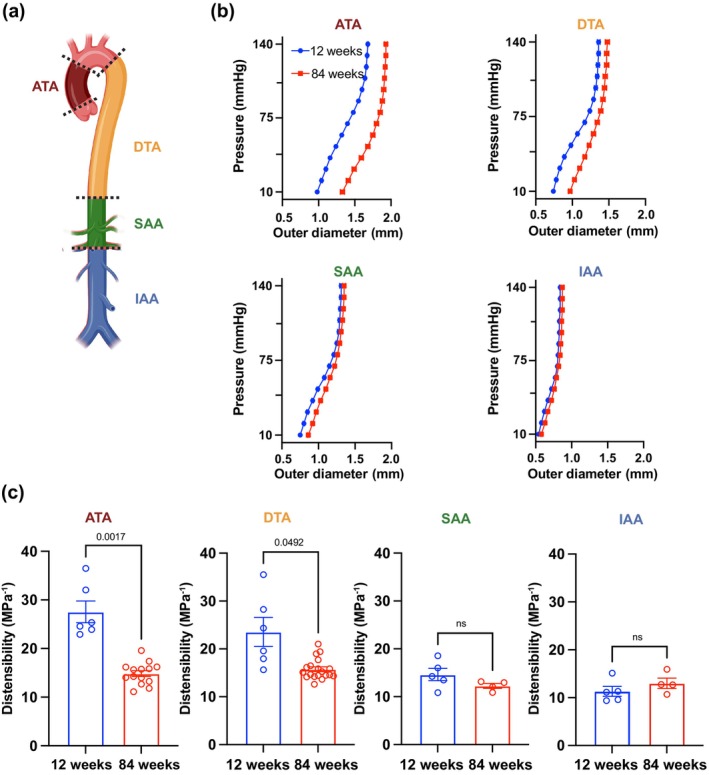
Spatial heterogeneity in the structural stiffening of the aorta during aging. (a) Schematic detailing the location of the four anatomical segments of interest from proximal to distal: ascending (ATA) and descending (DTA) thoracic, and suprarenal (SAA) and infrarenal (IAA) abdominal aorta. (b) Passive pressure vs. outer diameter response at 12 and 84 weeks of age in the four aortic regions. (c) Cyclic aortic distensibility in thoracic and abdominal segments at the 12 and the 84‐week endpoints. Statistical difference between age groups denoted by overbars with corresponding *p* values.

To evaluate whether different segments of the aorta age synchronously, we characterized the biaxial mechanical behavior of the ATA, descending thoracic aorta (DTA), suprarenal abdominal aorta (SAA), and infrarenal abdominal aorta (IAA) of young (12‐week‐old) and aged (84‐week‐old) mice (Figure 1a). The passive pressure versus diameter (P‐d) response of the aorta shifted toward larger diameters with age, though this effect progressively faded when moving away from the heart (Figure 1b). The change in the shape of the P‐d curve was more pronounced in proximal segments, consistent with an earlier engagement of collagen fibers and suggestive of decreased ability to deform within the physiological range of pressures (Ferruzzi et al., 2013) (Figure 1b). Indeed, the ATA experienced the largest decrease in cyclic distensibility, which remained significant but muted in the DTA, then subsided in the abdominal segments (Figure 1c). As our results demonstrate accelerated effects of aging on the ascending segment of the thoracic aorta, we will focus on this region for the rest of the work.

### 2.2 Mapping the unicellular transcriptome of the ATA during aging

**FIGURE 2:**
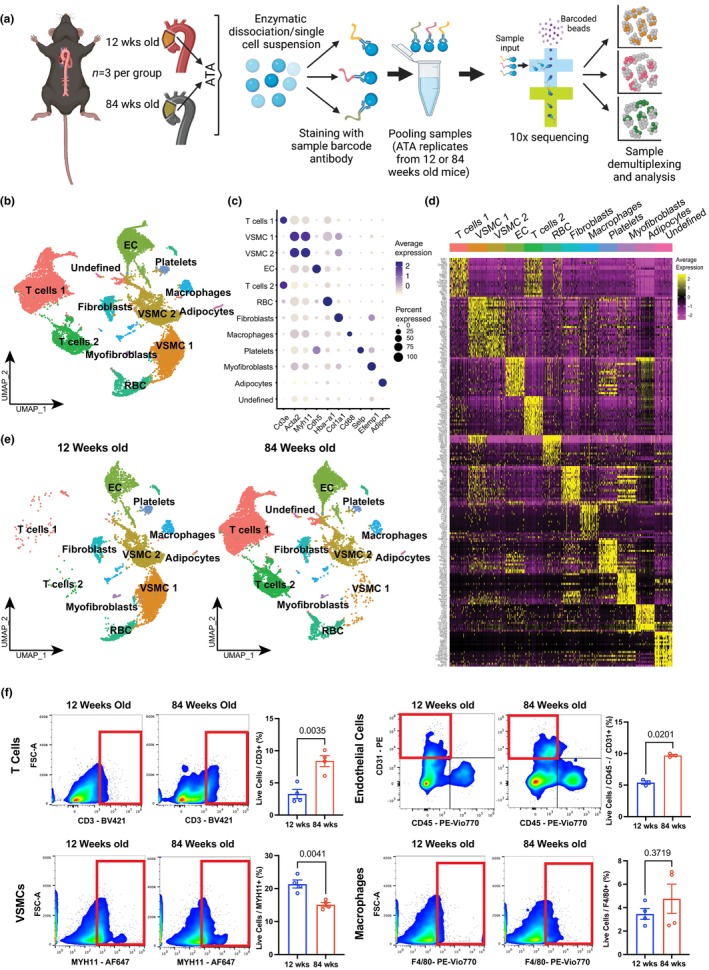
Mapping the unicellular transcriptome of the wildtype ATA during aging. (a) Schematic detailing sample collection, demultiplexing, and analysis. (b) Representative uniform manifold approximation and projection (UMAP) of cell clusters in the murine ATA. (c) Plot of cell clusters identified based on average expression of genes. (d) Heatmap of top differentially expressed transcripts by each cell clusters using expression of canonical markers to tag vascular cells. (e) UMAP of ATA cell clusters at the 12‐ and 84‐week endpoints. *n* = 3/sample/age group. (f) Representative flow cytometry plots showing the proportions of cell populations in the 12‐ and 84‐week‐old ATA. Data is quantified in corresponding bar charts of T Cells (CD3‐positive, upper left), vascular smooth muscle cells (Myh11‐positive, lower left), endothelial cells (CD31‐positive, CD45‐negative, upper right), and macrophages (F4/80‐positive, lower right). Data is represented as mean values ± SEM. *n* = 4/group/age. Statistical difference between age groups denoted by overbars with corresponding *p*‐values.

To unbiasedly catalogue the multicellular transcriptomic variations that arise during chronological aging of the ATA, we performed single‐cell RNA sequencing (scRNA‐seq) of ATA from 12‐ and 84‐week‐old mice, using the 10× Genomics platform. Three samples in each age group were digested and 15,000 cells from each of the 12‐ and 84‐week‐old ATAs were processed for integrated single‐cell sequencing to detect the transcriptome (Figure 2a). Quality control consisted of removing cells that exhibited more than 5% of mitochondrial DNA. Unsupervised Seurat clustering of the single‐cell data allowed identification of 12 aortic cell populations, as shown in the Uniform Manifold Approximation and Projection (UMAP) plots (Figure 2b). Top expressed genes per cluster discriminated each cell population (Figure 2c). We utilized a canonical set of markers (Table S1) represented in the heatmap (Figure 2d) to identify the clusters in the ATA—endothelial cells (EC) (*Pecam1*, *Cdh5*), vascular smooth muscle cells (VSMC) (*Acta2*, *Myh11*, and *Myl9*), and aortic fibroblasts (*Col1a1*, *Col3a1*, *Dcn*, and *Gsn*). Interestingly, there was an enrichment of immune cell populations characterized by T lymphocytes (*Cd3e*, *Cd8a*, *Trbc1*, and *Trbc2*) in aged ATA samples, and we observed a shift in the presence of vascular cells and T lymphocytes in the ATA with aging (Figure 2e). To characterize these cellular proportions, we performed flow cytometry analysis of young and aged ATA samples. We observed a ~2‐fold upregulation of T cells (CD3^+^) and EC (CD45^−^CD31^+^) but loss of VSMC in aged versus young ATA. Although we found a trend towards increased macrophages with age, the differences between young and aged tissues were not statistically significant (Figure 2f). These results suggest that chronological aging impacts the qualitative and quantitative immuno‐vascular phenotypes of the ATA.

### 2.3 Diverse patterns of signaling pathways during aging of the ATA

**FIGURE 3:**
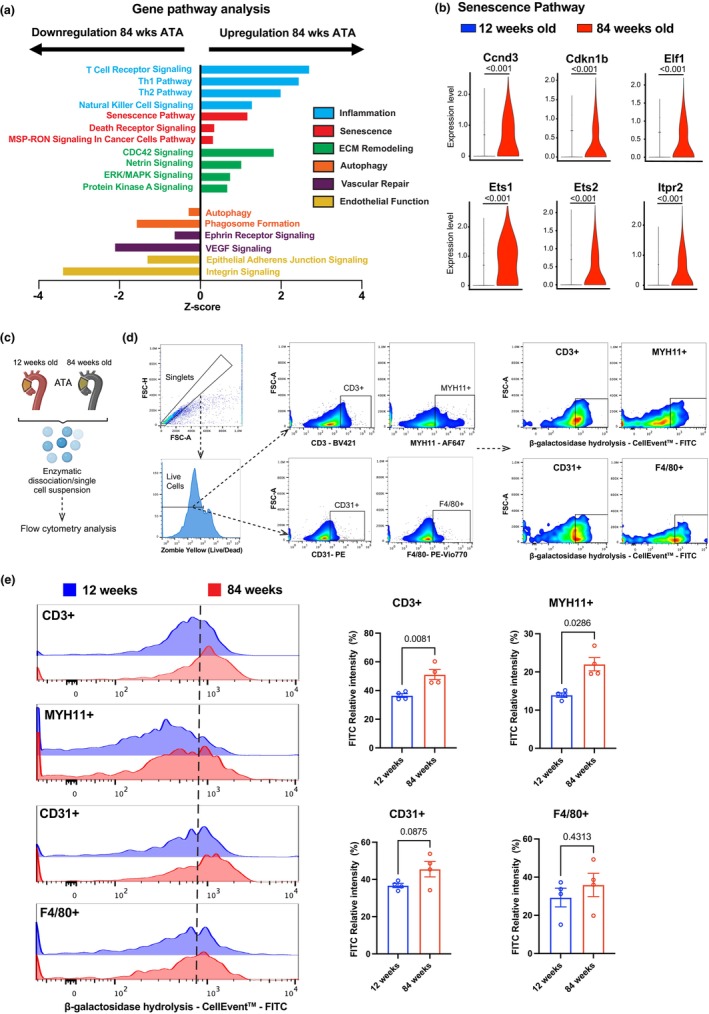
Diverse patterns of signaling pathways in the wildtype ATA during aging. (a) Up‐ and down‐regulated signaling pathways (IPA) in ATA tissues from 12 and 84‐week‐old mice. (b) Senescence pathway‐associated genes as indicated in the young and aged ATA. (c) Experimental workflow depicting ATA enzymatic dissociation and single‐cell suspension preparation for subsequent flow cytometry analysis. (d) Representative flow cytometry gating strategies, showing singlet cell isolation based on forward scatter height (FSC‐H) versus forward scatter area (FSC‐A) density plots, inclusion of only Zombie^+^ live (left panels) further gated to distinguish T Cells (Cd3‐positive), vascular smooth muscle cells (Myh11‐positive), endothelial cells (Cd31‐positive), and macrophages (F4/80‐positive). Right panels exhibit flow cytometric assessment of cellular senescence via β‐galactosidase hydrolysis using the CellEventTM Senescence Green probe for each identified cell population. (e) Representative overlay histograms showing FITC^+^ (senescence) relative intensity and quantification (right panel) for each cell population in the 12‐ and 84‐week‐old ATA. Data is represented as mean values ± SEM. *n* = 4/group/age. Statistical difference between age groups denoted by overbars with corresponding *p* values.

To delve into the molecular pathways that are altered during aging, we performed enrichment pathway analysis using the Ingenuity Pathway Analysis (IPA) software. We observed an overrepresentation of cellular senescence, ECM remodeling, and acquired immune response pathways in the ATA of 84‐ compared to 12‐week‐old mice (Figure 3a). In contrast, a decline in endothelial integrity, angiogenic responses, and autophagy was noted in aged compared to young ATA samples (Figure 3a). Elevated cellular senescence in aged tissues was denoted by upregulation of a panel of markers previously reported in the literature (Budka et al., 2018; Fry & Inoue, 2018; Jang et al., 2015; Jung et al., 2019; Lu et al., 2019; Tahara et al., 1995; Yu et al., 2018; Ziegler et al., 2021), namely *Ccnd3*, *Cdkn1b*, *Elf1*, *Ets1*, *Ets2*, and *Itpr2* (Figure 3b). Segregated distribution of cellular senescence per cluster by age shows accumulation of these genes in several cell types including T lymphocytes (Figure S1). To precisely map cell‐specific senescence in the young and aged ATA, unicellular homogenate of digested aortic tissues was stained with a cocktail of antibodies to detect distinct cell clusters (F4/80 for macrophages, CD31 for EC, CD3 for T lymphocytes, MYH11 for VSMC) and including the CellEvent senescence flow cytometry assay kit that enables detection of FITC^+^ cellular senescence via β‐galactosidase hydrolysis. We observed an increase in FITC^+^ signal in T lymphocytes and VSMC. There was a trend towards increased cellular senescence in EC and macrophages; however, differences were not statistically significant (Figure 3c–e). These results indicate that several canonical pathways of aging are present in the ATA and are likely to contribute to pathological vascular remodeling.

### 2.4 Heterogeneous accumulation of T lymphocytes during aging of the ATA

**FIGURE 4:**
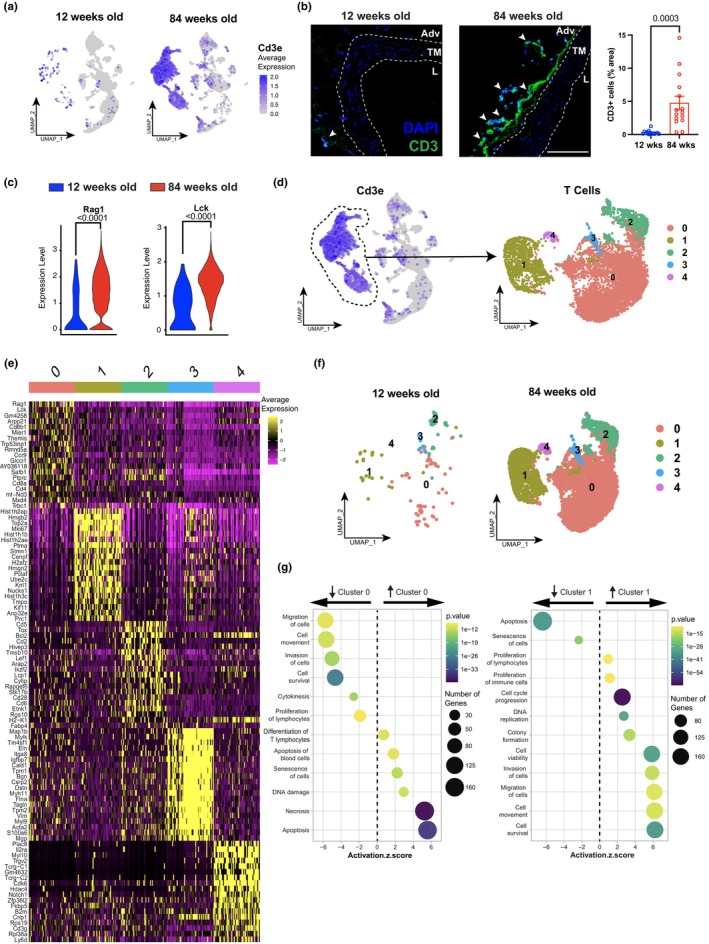
Heterogeneous accumulation of T lymphocytes in the wildtype ATA during aging. (a) UMAP visualization of Cd3e *mRNA* expression, for identification of distinct T lymphocyte cell clusters in ATA from 12‐ and 84‐week‐old mice. (b) Immunofluorescence staining of CD3 (green) and quantification (right panel) with DAPI (blue) in ATA aged as indicated. Tunica media (TM) is delineated between white dotted lines, Adventitial area (Adv), Lumen (L). White arrows indicate CD3‐positive. Scale bar is 100 μm (c) Expression profiles of Rag1 and Lck in the ATA aged as indicated. *n* = 3/group (d) UMAP showing re‐clustering of T lymphocytes into five sub‐clusters (0–4). (e) Heatmap displaying the top 20 differentially expressed transcripts for each sub‐cluster of T lymphocytes. (f) UMAP visualization of ATA T lymphocyte cell clusters between ages (g) Up‐ and down‐regulated signaling pathways in cluster 0 (left panel) and cluster 1 (right panel). Data is represented as mean values ± SEM. *n* = 14 square regions in 4 ATA sections for 12‐week‐old mice and *n* = 15 square regions in 5 ATA sections for 84‐week‐old mice. Statistical difference between age groups denoted by overbars with corresponding *p* value.

Since T cell receptor signaling is one of the most abundant signaling pathways in the aged ATA, we further dissected the nature of T lymphocyte distribution in ATA tissues. We identified distinct clusters of CD3 lymphocytes in our dataset (Figure 4a). Immunofluorescence analysis showed that CD3^+^ lymphocytes accumulated abundantly in the adventitia of the aged ATA (Figure 4b). We noted an enrichment of *Rag1*, a gene providing instructions for an essential component involved in the development and maturation of T and B cells (Mombaerts et al., 1992) and *Lck* (lymphocyte‐specific protein tyrosine kinase), a key regulator of T‐cell development, homeostasis, and TCR signaling (Bommhardt et al., 2019), within T lymphocytes in the aged ATA (Figure 4c). We re‐clustered the T lymphocyte population (unsupervised) which revealed segregation into five new subclusters (Figure 4d). A heatmap shows top expressed list of genes in each cluster (Figure 4e). Of note, *Rag1* and *Lck* were prominent in the dominant cluster 0. Several genes regulating histone family such as *Hist1h2ap*, *Hist1h1b*, *Hist1h2ae*, *Hist1h3c* as well as *Mki67* were highly expressed in cluster 1, suggesting enhanced chromatin remodeling and proliferation of the cells comprising this subset. Cluster 2 expressed a set of genes including *Cd5*, which has been shown to directly correlate with the affinity of TCR and actively regulates TCR pathways (Tarakhovsky et al., 1995). Segregation of the clusters by age revealed an expansion of all clusters in the aged ATA, with dominant representation of cluster 0 followed by cluster 1 (Figure 4f). Pathway analysis of cluster 0 showed that these cells were mostly anergic displaying apoptotic and senescent traits. T lymphocytes comprising the second most abundant cluster (cluster 1) exhibited more active phenotypes characterized by proliferation, DNA replication, and cell survival regulatory pathways (Figure 4g). Altogether, T lymphocyte signaling was broadly elevated in the aged ATA, revealing a key role for the adaptive immune response in the remodeling of the aortic wall during aging.

### 2.5 Microstructural matrix adaptations of the ATA wall during aging

**FIGURE 5:**
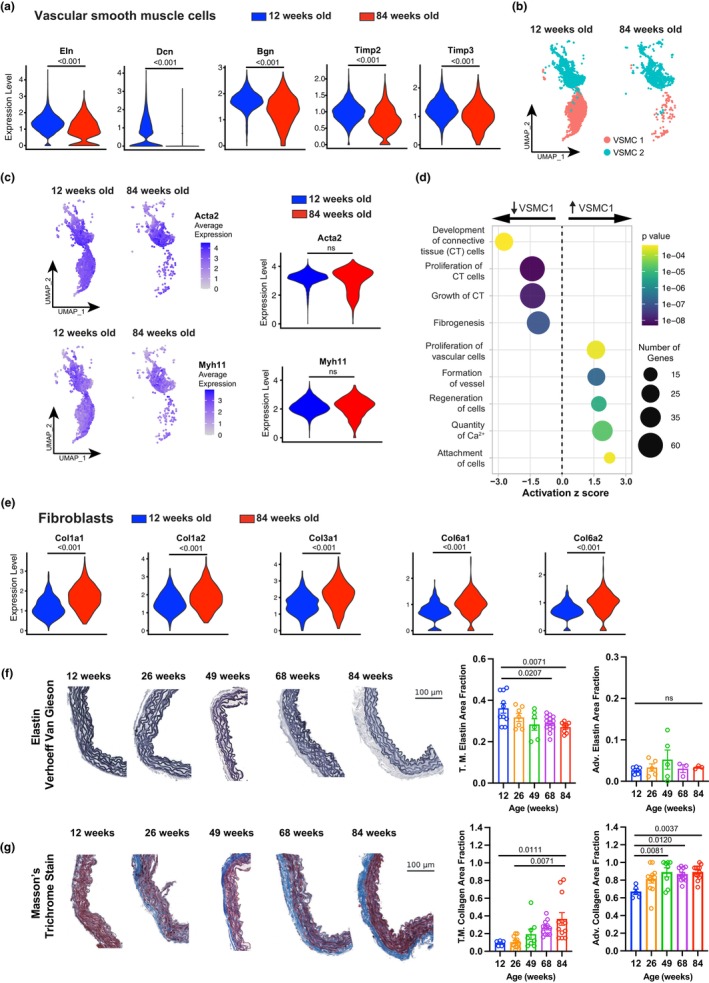
Microstructural matrix adaptations of wildtype ATA tissues during aging. (a) Expression of elastin (*Eln*), Decorin (*Dcn*), Biglycan (*Bgn*), and tissue inhibitors of matrix metalloproteinases 2 and 3 (*Timp2, 3*) genes in the VSMC cluster of ATA from 12‐ and 84‐week‐old mice. (b) UMAP showing the distribution of VSMC clusters 1 and 2. (c) expression and violin plot quantification of *Acta2* and *Myh11* in the two VSMC clusters. (d) Up‐ and down‐regulated signaling pathways in cluster VSMC1 versus cluster VSMC2. (e) Violin plots showing expression of collagen superfamily genes, including *Col1a1*, *Col1a2*, *Col3a1*, *Col6a1*, and *Col6a2* in fibroblasts in ATA samples (f). Quantification of elastin content in the tunica media (T.M.) and adventitia of ATA cross sections stained with Verhoeff Van Gieson (VVG) stain at 12, 26, 49, 68, and 84 weeks of age. *n* = 10 square regions in 4 ATA sections for 12‐week‐old mice, *n* = 7 square regions in 3 ATA sections for 26‐week‐old mice, *n* = 6 square regions in 3 ATA sections for 49‐week‐old mice, *n* = 13 square regions in 3 ATA sections for 68‐week‐old mice, *n* = 8 square regions in 4 ATA sections for 84‐week‐old mice. Data is represented as mean values ± SEM. (g) Quantification of collagen (blue) content in the tunica media (T.M.) and adventitia of ATA cross sections stained with Masson's Trichrome (MTC) stain at 12, 26, 49, 68, and 84 weeks of age. *n* = 10 square regions in 4 ATA sections for 12‐week‐old mice, *n* = 7 square regions in 3 ATA sections for 26‐week‐old mice, *n* = 6 square regions in 3 ATA sections for 49‐week‐old mice, *n* = 13 square regions in 4 ATA sections for 68‐week‐old mice, *n* = 8 square regions in 4 ATA sections for 84‐week‐old mice. Data is represented as mean values ± SEM. Statistical difference between age groups denoted by overbars with corresponding *p* value.

Several markers comprising subfamilies that safeguard the ECM were altered transcriptionally in the aged ATA. Amongst those, the expression of elastin (*Eln*), decorin (*Dcn*), biglycan (*Bgn*), and tissue inhibitors of matrix metalloproteinases 2 and 3 (*Timp2, 3*) declined within the VSMC of the aged ATA (Figure 5a). Notably, we observed two distinct populations of VSMC in the ATA (VSMC1, VSMC2), with a clear loss of VSMC1 but preserved VSMC2 during aging (Figure 5b). Projections of *Acta2* and *Myh11* transcripts indicated that these genes were equally represented in each age group (Figure 5c). Pathway analysis showed a concentration of genes regulating pathways such as proliferation and cell adhesion in VSMC1, suggesting that these functions may be altered in the aged ATA, since VSMC1 declines during aging. VSMC2 seemed to be more of a secretory phenotype which is conserved during aging (Figure 5d, Figure S2). Overall, phenotypical differences were not as drastic as described in pathological conditions (e.g., atherosclerosis), which are expected to trigger more aggressive alterations of the VSMC compared to the chronological aging of aortic tissues. We also observed upregulation of collagen superfamily elements such as *Col1a1*, *Col1a2*, *Col3a1*, *Col6a1*, and *Col6a2* in fibroblasts during aging (Figure 5e). To corroborate transcriptional inferences of these cell types, we determined the microstructural composition of the ATA wall at selected endpoints throughout the mouse adult lifespan (Figure S3) and found alterations in the overall makeup of tissue microstructure with age. Histological analysis revealed that the area fraction of medial elastin exhibited a significant negative correlation with age (*r*_s_ = −0.450, *p* < 0.01), despite no marked elastic fiber degradation (Figure 5f). Collagen content in the adventitia broadened significantly between 12 and 49 weeks of age (67 ± 3% vs. 89 ± 5% area fraction), after which it remained stable (Figure 5g). Similarly, the relative collagen content of the media expanded with age, but did so more gradually, rising from 9 ± 1% at 12 weeks to 36 ± 7% at 84 weeks (Figure 5g). A significant positive correlation emerged between increasing age and the area fraction of collagen in each wall compartment (*r*_s_ = 0.692, *p* < 0.01 for the media and *r*_s_ = 0.364, *p* < 0.01 for the adventitia; Figure 5g). Overall, these findings recognize collagen remodeling as a dominant feature of microstructural aging in the medial and adventitial layers of the ATA wall.

### 2.6 Shift toward dysfunctional passive ATA wall mechanics during aging

The remodeling endured by the ATA wall during aging may support a progressive shift toward a collagen‐driven mechanical response, consistent with the large and significant decline in cyclic distensibility we observed between 12 and 84 weeks of age. To establish a timeline for this process, we performed passive inflation/extension tests on ATA samples at the same endpoints considered for microstructural analysis (Figure S3). Note, mice steadily gained weight until 68 weeks, when their body mass reached a plateau (Table S2). Importantly, systolic and diastolic values of peripheral blood pressure remained constant throughout (Table S2).

**FIGURE 6:**
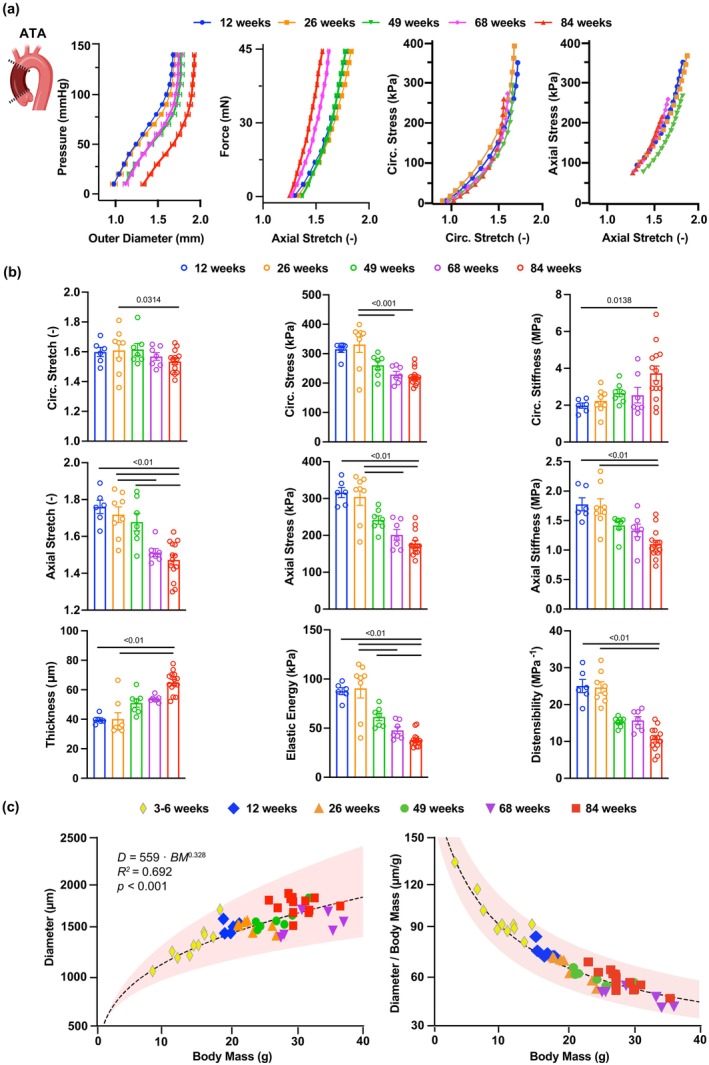
Progressive loss of mechanical functionality in the wildtype ATA during aging. (a) Average (±SEM) structural (pressure vs. outer diameter, axial force vs. stretch) and tissue (Cauchy stress vs. stretch) behavior of ATA samples in the circumferential and axial directions at 12, 26, 49, 68, and 84 weeks of age. (b) Biaxial descriptors of mechanical function in the ATA at the 5 endpoints. Metrics of stretch, stress, stiffness, thickness, and energy are calculated at group‐specific values of systolic pressure. Cyclic distensibility accounts for group‐specific values of luminal pressure and diameter between diastole and systole. (c) Allometric scaling (dashed line) of aortic size with body mass in ATA samples (symbols) throughout the mouse lifespan. Trends are reported for both absolute and normalized (by body mass) predictions of systolic luminal diameter. Red shaded area envelops the 95% confidence interval on estimated allometric parameters. Group sizes for the passive mechanical analysis are: 12 weeks (*n* = 6), 26 weeks (*n* = 8), 49 weeks (*n* = 8), 68 weeks (*n* = 9), and 84 weeks (*n* = 19). Note, we incorporated data from developing female wildtype mice (*n* = 9) to improve allometric predictions at low body mass. Statistical significance between individual age groups denoted by overbars with corresponding *p*values.

The passive response of the ATA gradually drifted with age. From a structural standpoint, a leftward shift in the axial force versus length behavior complemented the rightward shift in the pressure versus diameter curve (Figure 6a) and the upward shift in the volume versus pressure curve (Figure S4). Comparison of average biaxial stress versus stretch curves across groups further detailed the effect of aging on the intrinsic behavior of tissues, with progressive loss of circumferential distensibility and axial extensibility (Figure 6a). For completeness, Tables S3 and S4 report the best‐fit constitutive descriptors of the mechanical response of ATA tissues, and Table S5 includes related predictions of morphological and mechanical parameters. To facilitate visualization of aging‐related trends, geometrical, structural, and mechanical metrics are also plotted as a function of age in Figure S5.

The aorta dilated as mice aged (*r*_s_ = 0.618, *p* < 0.01), with the unloaded luminal diameter expanding by a factor of 1.2 from 892 ± 43 μm at 12 weeks to 1060 ± 25 μm at 84 weeks. Likewise, the unloaded thickness of the aortic wall increased from 111 ± 1 μm at 12 weeks up to 146 ± 5 μm at 84 weeks (*r*_s_ = 0.808, *p* < 0.01). Thickening and luminal widening persisted when evaluated at physiological levels of pressure and axial stretch. Systolic luminal diameter rose by a factor of 1.1 from 1560 ± 24 μm at 12 weeks to 1782 ± 22 μm at 84 weeks (*r*_s_ = 0.632, *p* < 0.01), while systolic thickness increased from 40 ± 1 μm to 65 ± 2 μm between 12 and 84 weeks (*r*_s_ = 0.759, *p* < 0.01; Figure 6b). Because representative (through‐thickness average) diameters when in the traction‐free configuration and under the effect of physiological loads varied in a similar manner during aging, the circumferential stretch did not significantly correlate with age and only exhibited a significant difference between 26 and 84 weeks (1.61 ± 0.05 vs. 1.53 ± 0.02; Figure 6b). Since wall thickening outpaced the widening of the lumen, circumferential wall stress showed a negative correlation with age (*r*_s_ = −0.574, *p* < 0.01), whereby it declined after 26 weeks toward significantly lower values at 68 and 84 weeks (331 ± 27 kPa at 26 weeks vs. 222 ± 7 kPa at 84 weeks; Figure 6b). Nevertheless, the intrinsic stiffness of tissues in the circumferential direction increased with advancing age (*r*_s_ = 0.521, *p* = 0.001), from 1.98 ± 0.14 MPa at 12 weeks to 3.73 ± 0.40 MPa at 84 weeks (Figure 6b, Figure S6).

The axial stretch steadily decreased with age (*r*_s_ = −0.738, *p* < 0.01), first becoming statistically different between 26 and 68 weeks (1.72 ± 0.04 vs. 1.51 ± 0.02; Figure 6b). Likewise, both axial stress (*r*_s_ = −0.707, *p* < 0.01) and axial tissue stiffness (*r*_s_ = −0.695, *p* < 0.01) displayed a strong negative correlation with age. The decline in stress again reached statistical significance between 26 and 68 weeks (from 304 ± 23 kPa to 188 ± 13 kPa; Figure 6b), while the decrease in stiffness was delayed to 84 weeks (from 1.74 ± 0.13 MPa to 1.10 ± 0.06 MPa; Figure 6b).

The cyclic distensibility of the wall decreased by a factor of 2.4 from 25.0 ± 1.8 MPa^−1^ at 12 weeks to 10.6 ± 0.8 MPa^−1^ at 84 weeks and was negatively correlated with age (*r*_s_ = −0.859, *p* < 0.01; Figure 6b). The elastic energy responsible for blood flow augmentation also exhibited a negative correlation with age (*r*_s_ = −0.792, *p* < 0.01), whereby the first significant decline occurred between 26 and 68 weeks (90 ± 10 kPa vs. 48 ± 4 kPa; Figure 6b) and continued thereon to the last endpoint (38 ± 2 kPa at 84 weeks). These findings indicate that the structural stiffening of the ATA happens gradually throughout the mouse lifespan and that aging drastically impairs the ability of the ATA to passively deform and recoil under pulsatile pressure loads, which combined contribute to vascular dysfunction with advancing age.

### 2.7 Effect of body mass on ATA diameter

Body mass imparted a significant effect on aortic caliber across age groups [F (6, 43) = 6.770, *p* < 0.001]. Predicted systolic luminal diameters scaled allometrically rather than isometrically with body mass. Estimated values for the coefficients that describe the exponential relationship between the two metrics are β=0.328 and $\alpha =559\;m\cdot g^{\beta }$ (*R* ^2^ = 0.692; Figure 6c). Because of this relationship, the ratio between the luminal diameter of the normal ATA and body mass was not constant, but rather varied with body mass (Figure 6c). Thus, normalization of aortic caliber by body mass should only be used as a comparison metric when no differences in body mass are observed across experimental groups (Farra et al., 2021).

### 2.8 Enhanced Piezo‐1 expression and ATA vasoconstriction during aging

**FIGURE 7:**
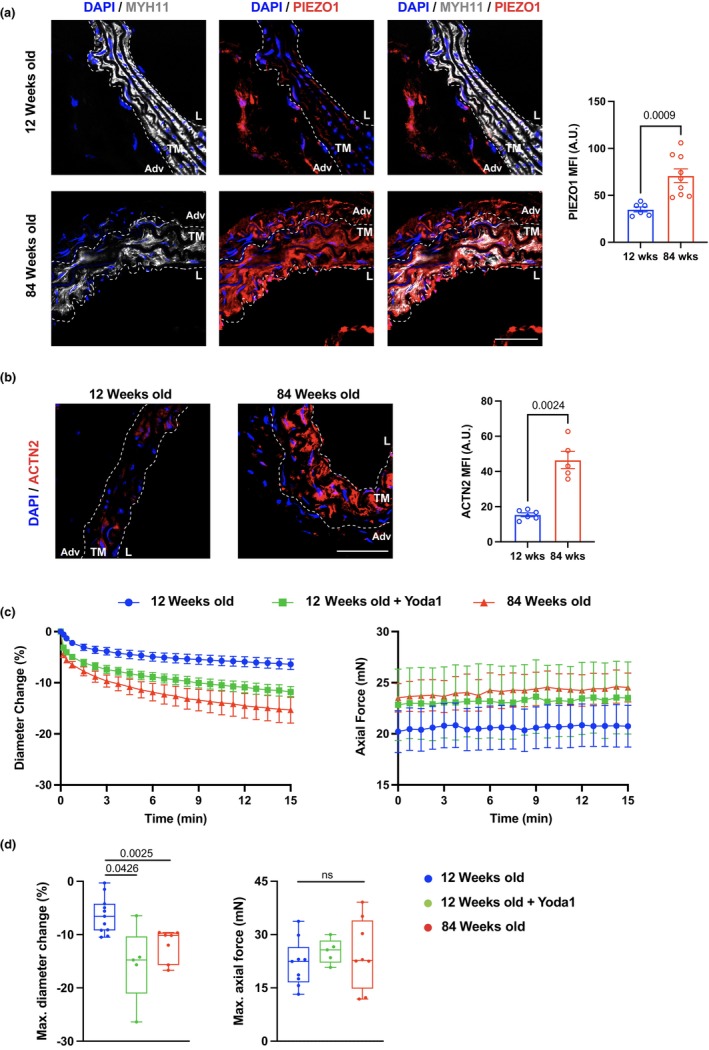
Overexpression of Piezo‐1 in aged ATA tissues and enhanced ATA vasoconstriction during aging in wildtype mice. (a) Representative microscopy image of immunofluorescent staining of Myosin heavy chain 11 (MYH11, white), Piezo‐1 (Piezo‐1, red), and DAPI (cell nuclei, blue) in 12‐ and 84‐week‐old ATA tissues. TM indicates the tunica media area outlined by white dotted lines, Adventitia (Adv), and L denotes the luminal edge of the vessel. Scale bar is 50 μm. Bar graph shows relative fluorescent intensity of Piezo‐1 (right panel). Data is represented as mean values ± SEM. *n* = 6 square regions in 4 ATA sections for 12‐week‐old mice and *n* = 9 square regions in 4 ATA sections for 84‐week‐old mice. (b) Representative microscopy image of immunofluorescence staining of α‐actinin 2 (ACTN2, red) and DAPI (cell nuclei, blue) in ATA aged as indicated, and quantification of intensity (right panel). Scale bar is 50 μm. Data is represented as mean values ± SEM. *n* = 6 square regions in 3 ATA sections for 12‐week‐old mice and *n* = 5 square regions in 3 ATA sections for 84‐week‐old mice. (c) Average (±SEM) traces of percent change in outer diameter and axial force following KCl stimulation of naïve ATA samples from 12‐ (*n* = 11) and 84‐ (*n* = 5) week‐old mice, and 12‐week‐old vessels preincubated with Yoda1 (*n* = 7). (d) Maximum change in diameter and axial force in response to KCl for naïve ATA samples at the 12‐ and 84‐week endpoints, as well as 12‐week‐old ATAs preincubated with Yoda1. Statistical significance between individual age groups denoted by overbars with corresponding *p* values.

Evidence of circumferential tissue stiffening during aging prompted us to assess tissue expression of one of the mechanosensitive ion channels, Piezo‐1, to explore the molecular mechanisms underlying the age‐related maladaptive remodeling of aortic tissues. Piezo‐1 upregulation has been shown in disparate cell types in conjunction with matrix stiffening (Atcha et al., 2021; Chen et al., 2018; Pathak et al., 2014; Wu et al., 2023) and we have documented Piezo‐1 engagement in stiffer aneurysmal tissues (Qian et al., 2022). Immunostaining of ATA tissues revealed marked upregulation of Piezo‐1 protein that mostly colocalized with Myh11 signal in the aged ATA (Figure 7a). Validation of VSMC‐specific anti‐Piezo‐1 antibodies was performed by staining of ATA sections in their presence or absence (Figure S7A). Additionally, neither of the antibodies captured VSMC Piezo‐1 signal in ATA sections from mice with conditional deletion of *Piezo‐1* in VSMC (Figure S7B). We further delved into the signals that upregulated Piezo‐1 in VSMC by probing for α‐actinin 2, as we have previously shown that this actin fiber crosslinker activates Piezo‐1 expression in VSMC, thereon modulating VSMC responses in aortic aneurysms (Qian et al., 2022). Similar to Piezo‐1, the expression of α‐actinin 2 was elevated in VSMC ensconced in the media of the 84‐week‐old ATA (Figure 7b), suggesting a co‐regulatory mechanism in VSMC. Noting that Piezo‐1 is a calcium‐permeable (Coste et al., 2010) and voltage‐sensitive (Moroni et al., 2018) ion channel, we compared the vasoactive ATA response to KCl at the 12‐ and 84‐week endpoints. We observed aging‐induced enhancement of ATA vasoconstriction under circumferentially isobaric (80 mmHg transmural pressure) and axially isometric (sample‐specific in vivo stretch) conditions reproducing physiological loads. The initial decline in outer diameter occurred more rapidly in ATA specimens from 84‐ compared to 12‐weeks‐old mice (Figure 7c) and the relative maximal vasoconstriction by the end of the 15‐min testing window was more pronounced in aged than young ATA samples (16 ± 3% vs. 6 ± 1% in magnitude at 84 and 12 weeks, respectively; Figure 7d). Furthermore, pharmacological activation of Piezo‐1 by Yoda1 in young ATA samples recapitulated the augmented vasoconstrictive response to KCl observed in aged samples (12 ± 1% magnitude of relative maximal vasoconstriction; Figure 7d). Neither increasing age nor Yoda1 treatment altered the axial force during vasoconstriction (Figure 7c, d).

### 2.9 VSMC‐derived Piezo‐1 partly mediates vasoconstriction and functional deterioration of the ATA during aging

**FIGURE 8:**
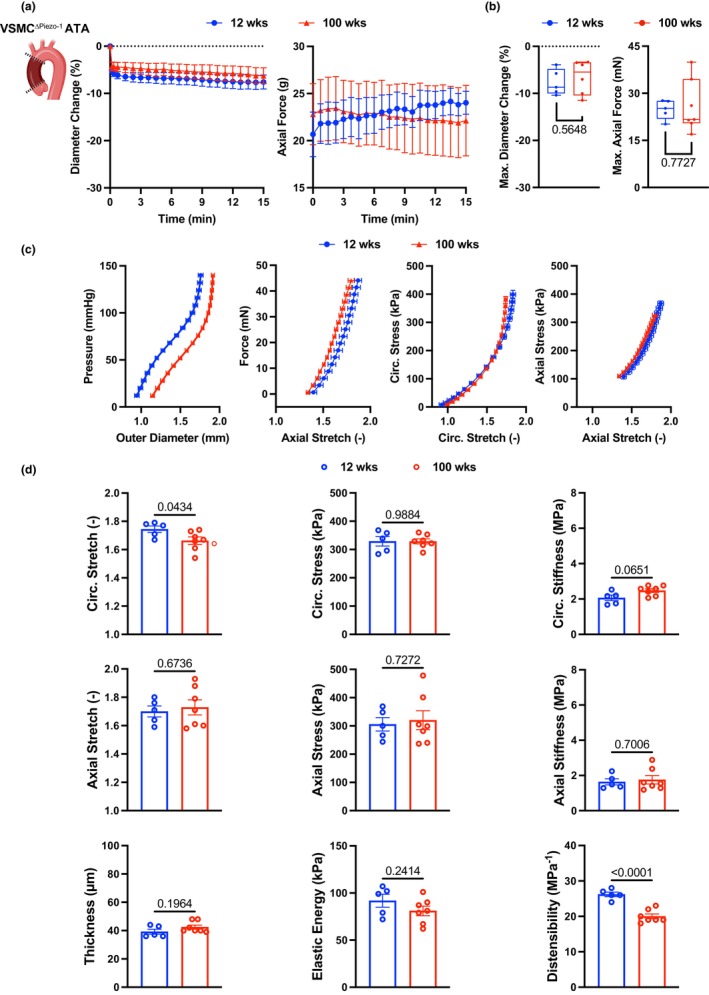
Loss of Piezo‐1 in VSMC prevents exaggerated vasoconstriction to KCl and partly rescues the functional decline of the aged ATA. (a) Average (±SEM) traces of percent change in outer diameter and axial force following KCl stimulation of ATA samples from 12‐ (*n* = 5) and 100‐ (*n* = 7) week‐old VSMC^ΔPiezo‐1^ mice. (b) Maximum change in diameter and axial force in response to KCl for VSMC^ΔPiezo‐1^ ATA samples at the 12‐ and 100‐week endpoints. (c) Average (±SEM) structural (pressure vs. outer diameter, axial force vs. stretch) and tissue (Cauchy stress vs. stretch) behavior of ATA samples from VSMC^ΔPiezo‐1^ mice in the circumferential and axial directions at 12 and 100 weeks of age. (d) Biaxial descriptors of mechanical function in the VSMC^ΔPiezo‐1^ ATA at the two endpoints. Metrics of stretch, stress, stiffness, thickness, and energy are calculated at group‐specific values of systolic pressure. Cyclic distensibility accounts for group‐specific values of luminal pressure and diameter between diastole and systole. Statistical significance between individual age groups denoted by overbars with corresponding *p* values.

The results above demonstrate that Piezo‐1 expression in aged ATA tissues predominantly localized in the VSMC‐populated media and identified pharmacological Piezo‐1 activation as a mechanism by which the percent vasoconstriction of the young ATA matched that of the aged ATA. Therefore, to corroborate the direct role of VSMC‐derived Piezo‐1 in augmenting ATA vasoconstriction during aging, we performed vasoactive tests on ATA samples from mice with conditional deletion of Piezo‐1 in VSMC (Piezo‐1^flox/flox^Sm22^Cre+^; VSMC^ΔPiezo‐1^). Compared to wildtype and independent of age, lack of Piezo‐1 in VSMC induced hastier ATA vasoconstriction upon KCl administration (Figure 8a). Following this initial dynamical phase, the percent decrease in diameter remained stable throughout the test (Figure 8a). Opposite to the age‐dependent vasoactive behavior of the wildtype ATA, the relative maximal vasoconstriction of the ATA was comparable in young and aged VSMC^ΔPiezo‐1^ mice (7 ± 1% vs. 8 ± 1% in magnitude at 100 and 12 weeks, respectively; Figure 8b). Again, age did not alter the time course (Figure 8a) nor the maximum value (Figure 8b) of axial force during vasoconstriction. These data indicate that the absence of Piezo‐1 in VSMC rescues the age‐related enhancement of ATA vasoconstriction to KCl and implicate Piezo‐1 engagement in mediating the augmented vasoactive response of the ATA during aging.

To explore the contribution of VSMC Piezo‐1 to the age‐induced maladaptive remodeling of aortic tissues, we further performed passive inflation‐extension tests on ATA samples from VSMC^ΔPiezo‐1^ mice. Body mass of VSMC^ΔPiezo‐1^ mice was higher at the 100‐week compared to the 12‐week endpoint, but systolic and diastolic values of peripheral blood pressure were comparable between the two age groups (Table S2). The biaxial behavior of the ATA as a conduit and of ATA tissues from VSMC^ΔPiezo‐1^ mice (Figure 8c) were qualitatively similar to those from wildtype mice of comparable age (Figure 6a), yet the loss of circumferential distensibility and axial extensibility were less pronounced. Best‐fit constitutive descriptors of the mechanical response of ATA tissues from VSMC^ΔPiezo‐1^ mice are listed in Table S3 and were used to predict morphological and mechanical parameters under *in vivo* loads reported in Table S6. Mirroring the widening of the lumen that occurs during aging in the wildtype ATA, the luminal diameter of the VSMC^ΔPiezo‐1^ ATA increased by a factor of 1.2 from 822 ± 8 μm at 12 weeks to 952 ± 29 μm at 100 weeks in the traction‐free state, and by a factor of 1.1 from 1596 ± 37 μm at 12 weeks to 1743 ± 34 μm at 100 weeks when pressurized and axially extended to replicate in vivo loads. Consistent with evidence of caudal artery thickening in response to Piezo‐1 signaling by VSMC (Retailleau et al., 2015), lack of VSMC Piezo‐1 preserved ATA wall thickness during aging in both the unloaded (115 ± 2 μm at 12 weeks vs. 122 ± 3 μm at 100 weeks) and pressurized (39 ± 2 μm at 12 weeks vs. 43 ± 1 μm at 100 weeks) configurations (Figure 8d). Maintenance of wall thickness permitted upholding circumferential stress in ATA tissues from VSMC^ΔPiezo‐1^ mice during aging (329 ± 17 kPa at 12 weeks vs. 329 ± 9 kPa at 100 weeks) despite lumen widening (Figure 8d). Akin to estimates for the wildtype aorta, ATA tissues from aged VSMC^ΔPiezo‐1^ mice experienced lower circumferential stretch compared to their young counterpart (1.75 ± 0.02 at 12 weeks vs. 1.66 ± 0.03 at 100 weeks; Figure 8d). Most importantly; however, loss of Piezo‐1 in VSMC prevented aging‐induced circumferential stiffening of ATA tissues (2.07 ± 0.16 MPa at 12 weeks vs. 2.48 ± 0.11 MPa at 100 weeks; Figure 8d). Similarly, axial stretch (1.70 ± 0.04 at 12 weeks vs. 1.73 ± 0.05 at 100 weeks), stress (306 ± 24 kPa at 12 weeks vs. 320 ± 33 kPa at 100 weeks), and stiffness (1.64 ± 0.17 MPa at 12 weeks vs. 1.76 ± 0.24 MPa at 100 weeks) in ATA tissues from VSMC^ΔPiezo‐1^ mice did not vary significantly with age (Figure 8d). Conservation of inherent biaxial stiffness in aged VSMC^ΔPiezo‐1^ ATA tissues contributed to limiting the age‐induced decline in cyclic distensibility to a factor of 1.3 between 26.3 ± 0.7 MPa^−1^ at 12 weeks and 20.0 ± 0.7 MPa^−1^ at 100 weeks (Figure 8d). Tissue capacity for energy storage serving as a surrogate metric for the Windkessel function of the aorta was also preserved in aged VSMC^ΔPiezo‐1^ mice (92 ± 7 kPa at 12 weeks vs. 81 ± 5 kPa at 100 weeks; Figure 8d). Collectively, these results suggest that Piezo‐1 acts as a critical gerogenic checkpoint by altering the structural/functional fate of the aorta during aging.

## 3 DISCUSSION

The consolidated relationship between vascular health and mortality (Virani et al., 2021) corroborates the existence of a molecular dialogue between aging and CVD (Lakatta et al., 2009; Le Couteur & Lakatta, 2010). Age‐related changes in the structure and function of the heart and blood vessels alter the milieu in which pathophysiological processes operate (Billman, 2020), influencing the threshold for disease inception, the rate of disease progression, and the severity of symptoms (Lakatta & Levy, 2003a). Likewise, pathological mechanisms may modify the natural course of the aging process, supporting a multi‐organ accelerated decay. Motivated by our interest in the superposition between aging and environmental pollutant inhalation (Eden et al., 2022; Farra et al., 2020, 2021; Matz et al., 2023), we probed cell makeup and gene/protein expression in tissues from the ATA of young and aged mice, to interpret the evolving geometrical features and functional metrics that this aortic segment exhibits throughout the mouse adult lifespan.

Alongside impairment of endothelium‐dependent dilatation in resistance arteries (Lakatta & Levy, 2003b; Matz et al., 2000; Virdis & Taddei, 2016), structural stiffening of the aorta has emerged as a mechanical signature of aging across species, from mice (Ferruzzi et al., 2018; Hawes et al., 2020; Rammos et al., 2014; Wheeler et al., 2015), rats (Lindesay et al., 2016), and rabbits (Cooper et al., 2012) to non‐human primates (Babici et al., 2020) and humans (Hopper et al., 2021; O'Rourke & Hashimoto, 2007). We confirm here that cyclic distensibility declines in the murine ATA between 12 and 84 weeks of age (Figures 1c, 6b; Table S5) and further investigate the interplay between hemodynamics, geometry, and tissue stiffness through intermediate endpoints (Hawes et al., 2020; Lakatta & Levy, 2003a). Besides wall thickness, both the luminal and outer diameters of the ATA progressively grow larger with age (Ferruzzi et al., 2018; Hawes et al., 2020; Wheeler et al., 2015) (Figure 6a; Table S5). Aortic caliber is primarily regulated by volumetric blood flow (Humphrey, 2008) and varies allometrically with body mass (Korneva et al., 2019). While we did not measure cardiac output, the coefficients of allometric scaling that we estimated from predicted luminal diameters at physiological pressure (Figure 6c) show good agreement with those reported in previous studies (Goergen et al., 2007; Korneva et al., 2019; Prim et al., 2018).

Progressive wall thickening and lumen widening of the aging ATA rely on collagen deposition and remodeling (Ferruzzi et al., 2018; Fleenor et al., 2010; Hawes et al., 2020; Wheeler et al., 2015) (Figure 5g). Our analysis indicates that these processes are not limited to the adventitia but also involve the media despite preserved elastic fiber integrity (Figure 5f). It has been shown that increasingly abundant collagen transitions to thicker and less undulated fibers in the adventitia and broadens the separation of adjacent elastic laminae in the media within the first year of the mouse life (Cavinato et al., 2021). Aligned with this evidence, we further report that collagen deposition in ATA tissues continues up to 84 weeks of age (Figure 5g), complementing reduced medial elastin content (Figure 5f). Note, although not fragmented (Figure 5f), elastic fibers within central arteries of mice may still lose mechanical competency with age due to increased enzymatic activity of elastase (Fleenor et al., 2010; Liu et al., 2015). As a result of these layer‐specific processes, mechanical loads are likely transferred to newly deposited/remodeled collagen fibers (or to stiffer smooth muscle bundles), thus promoting circumferential stiffening of medial and adventitial tissues within the physiological range of loads. We show that, compounded by the loss of axial extensibility, the diffuse circumferential stiffening of ATA tissues during aging impairs aortic function by reducing the elastic energy available for diastolic blood flow augmentation (Figure 6b; Table S5). These findings reveal new and unappreciated adaptations of ATA tissues imposed by chronological aging, and recognize imbalances in the redistribution of medial/adventitial microstructural proteins as characteristic traits of aging. It is likely that age‐dependent patterns of structural ECM elements infer a maladaptive state that tolerates disease onset.

Substantial accumulation of T‐lymphocytes in the aged ATA (Figure 4) upholds mounting evidence of immune system involvement in adventitial stiffening of central arteries during aging (Grabner et al., 2009; Moos et al., 2005). It was previously reported that accumulation of activated T‐cells enhances adventitial collagen deposition via cytokine release and stiffens thoracic aortic tissues from 6‐ and 9‐month‐old male mice with chronic vascular oxidative stress (Wu et al., 2016), a prominent feature of aging (Durrant et al., 2009; Lesniewski et al., 2009). Notably, T lymphocyte depletion significantly lowered aortic PWV and ameliorated mesenteric endothelium‐dependent dilatation in ~23‐month‐old male C57BL/6 mice (Trott et al., 2021). It was also shown that T cell infiltration in the adventitia of the thoracic aorta of male B6D2F1 mice aged ~30 months contributed to elevated pro‐inflammatory cytokines in the tissue (Lesniewski et al., 2011). These studies point to a role of T lymphocytes in regulating both stiffness and inflammation, either directly or through the interaction with other aortic/immune cells. Indeed, the cross‐talk between T cells and VSMC or fibroblasts has been shown to occur through various signaling pathways. T lymphocyte‐derived cytokines like interleukin‐17A have been implicated in protecting against angiotensin II‐induced aortic wall stiffening and adventitial fibrosis (Majesky, 2015). Furthermore, T cells secrete pro‐inflammatory cytokines such as TNF‐α and IFN‐γ, which activate aortic cells. This activation leads to the secretion of ECM proteins, contributing to vascular stiffness and fibrosis (Wu et al., 2016). Further research is needed to delineate the specific subtypes of T lymphocytes involved in the regulation of vascular stiffness. This will provide a more comprehensive understanding of the immune‐vascular interactions in the context of vascular aging.

Lack of T‐cell accumulation substantiates medial immune‐privilege (Tellides & Pober, 2015) and shifts the mechanistic burden for the age‐related stiffening of this aortic compartment onto VSMC. Depletion of VSMC in the 84‐week‐old ATA (Figure 2f, 5b) corroborates histological evidence showing progressive decrease in their area fraction during aging (Ferruzzi et al., 2018; Wheeler et al., 2015). Nevertheless, preserved myosin heavy chain 11 (MYH11) transcripts (Figure 5c) and fluorescence intensity in the media (Figure 7a) alludes to the retention of a contractile phenotype by either VSMC subsets (Figure 5b) during aging. Although one of the VSMC sub‐populations declines in aged ATA tissues, we did not observe striking differences in the pattern of gene expression (Figure 5c,d). Interestingly, comparison of the EC between the young and aged ATA also does not show drastic changes in expression profiles (Figure S8), despite doubling of the EC proportion during aging. This likely invokes that chronological aging imposes more nuanced effects on the EC and VSMC compared to those accompanying aortic diseases such as atherosclerosis. Nevertheless, ATA tissues exhibit circumferential stiffening and enhanced vasoconstriction at the 84‐week endpoint. This prompted evaluation of mechanosensitive Piezo‐1 as a candidate mediator of age‐related remodeling in the ATA media. We have recently shown that actin fiber crosslinking and cytoskeletal stiffening by α‐actinin 2 facilitate Piezo‐1 opening in VSMC, thereby promoting Ca^2+^ influx and proteolytic enzyme release in the aneurysmal abdominal aorta (Qian et al., 2022). Tissue expressions of Piezo‐1 and α‐actinin 2 significantly intensify in the VSMC‐populated media of the aged ATA (Figure 7a,b), supporting their involvement in the age‐induced functional decline of aortic tissues. Indeed, rescue of aberrant vasoconstriction in aged ATA samples from VSMC^ΔPiezo‐1^ mice (Figure 8a,b) admits a causative role for Piezo‐1 activation in aging‐induced aortic dysfunction. Importantly, loss of VSMC Piezo‐1 prevents wall thickening, circumferential tissue stiffening, and loss of capacity for energy storage in the aged ATA, curbing the decline in cyclic distensibility (Figure 8d). Further reinforcing this evidence, young ATAs treated with Yoda‐1 recapitulate the enhanced vasoconstriction exhibited by aged samples (Figure 7c,d), suggesting that Piezo‐1 activation and its signaling pathways are likely critical in regulating age‐mediated alterations in ATA function. Aligned with our findings, it was reported that Piezo‐1 activation by Yoda1 induces contraction of human pulmonary arterial VSMC and dose‐dependent vasoconstriction of rat intrapulmonary arteries, due to intracellular Ca^2+^ increase (Liao et al., 2021). These observations fuel maturing evidence of augmented calcium signaling (Stasiak et al., 2020) and contractility (Jamieson et al., 2021; Polio et al., 2019) in VSMC embedded within a stiff matrix. Note, Piezo‐1 signaling was shown to promote thickening of the murine caudal artery by stimulating the Ca^2+^‐dependent catalytic activity of transglutaminases (Retailleau et al., 2015) and tissue transglutaminase contributes to vascular stiffening during aging (Santhanam et al., 2010; Steppan et al., 2017; Wang et al., 2021). Therefore, modulation of the actin cytoskeletal architecture in VSMC may heighten contractility and contribute to ECM stiffening through the activation of mechanosensitive Piezo‐1 in the ATA media during aging. Surprisingly, however, *Piezo1* did not emerge as one of the top differentially expressed genes in the cell‐specific analysis of scRNA‐seq data in aged ATA tissues, although significant overexpression of the *Piezo1* gene was observed in the pan‐expression analysis. Nevertheless, given the strong evidence supporting the effect of VSMC Piezo‐1 on aortic function during aging, we speculate that *Piezo1* transcription may have occurred before the 84‐week endpoint. It is also important to note that several other cell types express Piezo‐1, including EC, T lymphocytes and macrophages. In this work, we have not investigated Piezo‐1 signaling in cell types other than VSMC, given its major role in mediating vasoconstriction of aortic tissues. Future studies are needed to evaluate the contribution of non‐VSMC Piezo‐1 activity during aortic aging.

While our study provides novel insights into the complex mechanisms of vascular aging, there are limitations to be considered. How our findings could be applied to the aging of human aortic tissues was not explored. Of note, the study of vascular aging in humans is limited due to unfeasibility (e.g., access of young healthy aortic tissues). Mouse models thus represent an alternative and powerful tool to delve into the complexities of vascular aging. Nonetheless, to translate information learned from murine models to human biology, it is critical to account for differences between the mouse and human cardiovascular systems. Amongst those, the life span of the human but not the mouse exceeds the half‐life of elastin. As a result, the age‐related microstructural remodeling of murine arteries does not involve overt elastic fiber fragmentation (as observed in humans) and relies primarily on collagen deposition/fibrosis (Hopper et al., 2021). Furthermore, pressure amplification as predicted in humans but not mice may stem from upright posture. These are important considerations when attempting to translate murine aortic mechanobiology to humans.

Overall, age remains one of the dominant risk factors for CVD, and our findings indicate that chronological aging is sufficient to impose adverse remodeling on aortic tissues, with traits favoring the development of vasculopathies such as aortic aneurysms and dissections. Our study reveals that aging alters the intrinsic mechanical and cellular properties of ATA tissues. This is an important feature clinically, as many patients suffering from cardiovascular ailments are advanced in age. Therefore, unraveling the biomechanics and mechanobiology of aging in aortic tissues is expected to ameliorate the clinical management of cardiovascular patients. Furthermore, we speculate that supplementing Piezo‐1 antagonists might improve the outcome of healing and recovery following aortic surgery.

In conclusion, we showed that chronological aging of the aorta is a complex process involving layer‐specific multicellular and tissue micro‐adaptations that manifest as a region‐dependent and progressive loss of function. We demonstrated that the structural stiffening is heterogeneous along the aortic tree, with the ATA exhibiting the most stringent decline in distensibility. We then separated molecular mechanisms that may independently support the collagen‐driven stiffening of the medial and adventitial layers of the wall, respectively. We also demonstrated that Piezo‐1 contributes to the aging of medial VSMC and impacts tissue remodeling. These new results further refine our understanding of the mechanisms that advance chronological vascular aging and provide a candidate platform of signals that could be investigated in ailments affecting the aorta.

## 4 MATERIALS AND METHODS

### 4.1 Animals and physiological measurements

All animal procedures adhered to NIH guidelines and received approval from the Institutional Animal Care and Use Committee (IACUC) at Northeastern University or New York University School of Medicine. Female C57BL/6 wildtype mice were purchased from Jackson Laboratory (Bar Harbor, ME) at 7–8 weeks of age and housed in groups of five under standard 12:12‐h light–dark cycle, with ad libitum access to water and chow. Mice were allowed to age up to either 12 ± 1, 26 ± 1, 49 ± 2, 68 ± 1, or 84 ± 4 weeks by random assignment. These endpoints cover most of the mouse adult lifespan and precede the stark age‐related decline in survival beyond 100 weeks of age (Yuan et al., 2015). Piezo‐1^flox/flox^ (Strain #:029213, Jackson laboratory) were bred with SM22Cre^+^ mice (Strain #:017491, Jackson laboratory) to generate Piezo‐^1flox/flox^Sm22Cre^+^ mice with conditional deletion of Piezo‐1 in vascular smooth muscle cells. Mice were allowed to age up to either 12.7 ± 0.1 weeks or 100.8 ± 0.5 weeks by random assignment. Body mass and tail‐cuff peripheral pressure (CODA, Kent Scientific) were measured in a subset of mice at endpoint, before CO_2_ euthanasia.

### 4.2 Single‐cell RNA sequencing

Ascending Thoracic Aorta (ATA) sections were obtained from young (12 ± 1 week‐old, *N* = 3) and aged (84 ± 4‐week‐old, *n* = 3) wildtype mice for single‐cell RNA sequencing (scRNA‐seq). Tissues were digested for 60 min at 37°C in an enzymatic mix composed of type II collagenase (10 mg/mL; C6885, Sigma Aldrich) and elastase (1 mg/mL; LS002292, Worthington Biochemistry), as previously described (Boytard et al., 2020; Qian et al., 2022). Single cell suspensions were obtained after passage of the digested lysate through a 70 μm cell‐strainer. Following digestion, viable cells were enriched using a dead cell removal magnetic kit (130‐090‐101, Miltenyi Biotech) and cellular viability was assessed using Trypan blue staining (0.4%; 1450013, Bio‐Rad) and an automated cell counter (TC20, Bio‐Rad). Viable cells (15 × 10^3^ per sample) were loaded on a 10× Genomics Chromium instrument to obtain individual gel beads in emulsion (GEMs). Library constructions were prepared using Chromium Single Cell 3′Reagent Kits v.2 (10× Genomics; PN‐120237, PN‐120236, PN‐120262).

The HiSeq 4000 (Illumina) was used for sequencing with 2 × 150 paired‐end reads (>90% sequencing saturation). Cell Ranger Single Cell Software Suite (version 3.1) was used to perform de‐multiplexing, barcode and unique molecular identifier (UMI) processing, and single‐cell 3′ gene counting (https://support.10xgenomics.com/single‐cell‐gene). Data analysis was performed using the Seurat sequencing package (version 4.0.5), R Studio Desktop (version 1.2.5033), and R (version 3.0.1+). Quality control, metrics, data normalization, scaling, batch correction, and dimension reductions were all performed using the Seurat package. The mitochondrial content was regressed out with the function SCT transform of the Seurat package, and neighboring and clustering were performed on the most significant principal component analysis.

### 4.3 Active biaxial mechanical testing

Immediately after sacrifice of young (12 ± 1 week‐old, *N* = 11) and aged (84 ± 4 week‐old, *N* = 5) wildtype or young (12.7 ± 0.1 week‐old, *N* = 5) and aged (100.8 ± 0.5 week‐old, *N* = 7) VSMC^ΔPiezo‐1^ mice, ATA specimens were excised and immersed in Hanks buffered salt solution (HBSS) chilled to 4°C. Excess perivascular tissue was quickly removed and lateral branches were ligated using one of three strands of a braided 9–0 nylon suture. Vessels were secured onto a stage using custom glass cannulae and immersed in a 37°C Krebs–Ringer's buffer solution, bubbled with a 95% O_2_–5% CO_2_ mixture to maintain pH at 7.4. The stage was mounted onto a computer‐controlled custom biaxial tester that applies luminal pressure and axial stretch while measuring outer diameter and axial force. Following preconditioning (40 mmHg pressure at 1.1 axial stretch + 60 mmHg pressure at 1.2 axial stretch), refinement of initial measurements of unloaded dimensions facilitated estimation of the in vivo axial stretch. Vessels were exposed to an 80 mmHg distending pressure while maintained at in vivo axial stretch and allowed to equilibrate. A subset of 12‐week‐old wildtype specimens (*N* = 7) was incubated for 30 min with 20 μM Yoda1 to activate Piezo‐1 mechanosensitive channels. Upon stabilization of outer diameter and axial force, vasoconstriction was induced by addition of a 40 mM KCl to the bath, which was washed out with fresh Krebs‐Ringer's solution 15 min later. Vasoconstriction throughout was expressed as a percent change in outer diameter,

(1) $$∆d_{o}\left(\%\right)=\frac{d_{o}-D_{0}}{D_{o}}\cdot 100,$$

where, $d_{o}$ is the current outer diameter and $D_{0}$ is the initial outer diameter prior to KCl treatment.

### 4.4 Passive biaxial mechanical testing

The custom biaxial device used for probing KCl vasoconstriction also facilitated passive mechanical testing, according to experimental protocols and analytical methods described elsewhere (Bellini et al., 2014; Bersi et al., 2016; Farra et al., 2021). Briefly, following dissection, removal of perivascular tissue, and ligation of lateral branches, each wildtype aorta was sectioned into ascending (ATA, *N* = 43) and descending (DTA, *N* = 50) thoracic and suprarenal (SAA, *N* = 10) and infrarenal (IAA, *N* = 10) abdominal segments, while the ATA (*N* = 12) segment alone was excised from VSMC^ΔPiezo‐1^ mice. Cannulated specimens were axially stretched and preconditioned under physiological pulsatile loads while submerged in room temperature HBSS to ensure a near passive mechanical behavior (Ferruzzi et al., 2011). Upon refinement of preliminary unloaded dimension and in vivo axial stretch estimates, the pressure‐diameter (P‐d, at varying axial stretch) and force‐length (F‐l, at varying pressure) responses were recorded through a set of 7 cyclic testing protocols.

### 4.5 Transmurally‐averaged mechanical properties

Experimental data were supplied to a nonlinear regression algorithm to fit a microstructurally motivated four‐fiber family strain energy potential (W) in the form

(2) $$W\left(C\right)=\frac{c}{2}\left(I_{C}-3\right)+\sum_{j=1}^{4}\frac{c_{1}^{j}}{4c_{2}^{j}}\left\{\exp\left[c_{2}^{j}{\left({\mathrm{IV}}_{C}^{j}-1\right)}^{2}\right]-1\right\},$$

where, $I_{C}$ and ${\mathrm{IV}}_{C}^{j}$ are the first and fourth invariants of the right Cauchy‐Green deformation tensor C and index j encompasses the axial (j=1), circumferential (j=2), and two axially symmetric diagonal (j=3,4) directions. The first isotropic Neo‐Hookean term (coefficient c, dimension of a stress) in Equation 2 accounts for the contribution of elastic fibers and amorphous matrix, while the Fung‐type terms (coefficients $c_{1}^{j}$ with the dimension of a stress and unitless coefficients $c_{2}^{j}$) describe the anisotropic response of collagen and smooth muscle bundles (Fung & Biomechanics, 1981). Best‐fit parameters were used to predict geometry and transmurally‐averaged tissue properties under desired pressure and axial loads, leveraging the small‐on‐large theory to determine the circumferential and axial components of linearized stiffness about the set working point within the cardiac cycle (Baek et al., 2007). Based on these predictions, the structural stiffness metric of cyclic aortic distensibility (D) was calculated from the systolic and diastolic values of blood pressure (P) and inner diameter ($d_{i}$), as:

(3) $$D=\frac{d_{i,\mathrm{sys}}-d_{i,\text{dias}}}{d_{i,\text{dias}}\;\left(P_{\mathrm{sys}}-P_{\text{dias}}\right)}.$$

### 4.6 Allometric scaling

Logarithmic transformation and linear regression of estimated systolic dimensions were used to yield best fit values for the α and β coefficients of the allometric scaling

(4) $$d_{i}=\alpha \;M^{\beta }$$

that relates luminal aortic diameter ($d_{i}$) to body mass (M) in wildtype mice.

### 4.7 Histology and immunofluorescence

Following completion of mechanical testing, wildtype ATA tissues reserved for histology were fixed overnight in 10% neutral buffered formalin and stored long‐term in 70% ethanol. Paraffin‐embedded samples were serially sliced into 5 μm thick cross sections that started ~500 mm distally of the aortic root. Once deparaffinized, cross sections were processed for tissue rehydration and antigen retrieval using EDTA and Tween solution. Tissues were stained with Verhoeff Van Gieson (VVG) or Masson's Trichrome (MTC) stains. Images of stained cross sections were acquired individually with a 20× objective. The area fractions of elastin and collagen were quantified by automatic thresholding of pixels based on hue (H, 0–360), saturation (S, 0–1), and lightness (L, 0–1) values specific to each stain (Bersi et al., 2016; Farra et al., 2021). Following removal of cell nuclei and debris, all pixels with lightness below 0.30 in VVG‐stained tissues were counted as elastin. Likewise, blue pixels (H 160–265, S 0.23–1.00, L 0.38–1.00) were considered as collagen in MTC‐stained cross sections.

Additional wildtype ATA samples were dissected and immediately embedded in optimal cutting temperature (OCT) compound (#4585, Fisher Scientific) and fast‐frozen for cryosectioning. Tissues were sliced into 7 μm thick cross‐sections and staining was performed by overnight incubation with primary antibodies: anti‐CD3 (ab11089, Abcam, 1:100 dilution), anti‐α‐actinin2 (14221‐1‐AP, Proteintech, 1:200 dilution), anti‐actin‐α‐2 (48938S, Cell Signaling, 1:200 dilution), anti‐MYH11 (ab224804, Abcam, 1:50 dilution), anti‐Piezo1, (15939‐1‐AP, Proteintech and APC‐087, Alomone labs, 1:200 dilution each). Alexa Fluor 488 and 568 conjugated anti‐IgG antibodies (Invitrogen) were used for fluorescent signal detection (1:500 dilution each). 4′,6‐diamidino‐2‐phenylindole (DAPI; Invitrogen) allowed visualization of the nucleus (1:5000 dilution). Images were acquired at ×40 magnification using a Zeiss LSM 710 confocal microscope (Carl Zeiss) and the Zeiss Efficient Navigation (ZEN) software (Carl Zeiss). Identical acquisition parameters were set to capture images from all age groups. Mean fluorescent intensity per unit area was computed using ImageJ software (NIH).

### 4.8 Flow cytometry and senescence detection

ATAs were collected and digested for 1 h at 37°C in an enzymatic mix composed of 10 mg/mL collagenase type II (#C6885, Sigma Aldrich) and 1 mg/mL elastase (#LS002292, Worthington Biochemistry). Digested aortas were mechanically disrupted and passed through a 70 μm cell strainer (BD Bioscience), centrifuged for 10 min at 1000 × g at room temperature. Cells were stained with Zombie Yellow^TM^ dye solution (1:100) in 1X PBS to a concentration of 1 × 10^6^ cells per 100 μL for 20 minutes at room temperature in the dark. After washing, a volume of 100 μL of aortic cell suspension was then stained with antibodies‐PE‐REAfinity™ anti‐CD31 (130‐111‐540, Miltenyi biotechnology), PE‐Vio^TM^ 770 anti‐F4/80 (130–118‐459, Miltenyi biotechnology) and BV421™ anti‐CD3 (100227, Biolegend), anti‐MYH11 (ab224804, abcam) 1:50 dilution each in 2%BSA for 20 minutes at room temperature, protected from light. Secondary antibody Alexa Fluor 647™ 1:100 dilution was used to detect MYH11. Stained cell suspension was fixed with PFA 2% in PBS for 10 min. An aliquot of unstained sample was used as control to determine the negative population. Only live cells were included in the analysis, defined as a negative for Zombie Yellow. Vascular smooth muscle cells were identified as MYH11‐positive cells in the density plot. T cells were identified as CD3‐positive cells in the density plot. Macrophage population was identified as F4/80‐positive cells in the density plot. Endothelial cells were identified as CD31‐positive and CD45‐negative cell populations. For senescence detection, washed cells were resuspended in CellEvent™ Senescence Green Probe (Thermo Fisher Scientific) (1:500 dilution), and incubated for 2 h in the dark. Samples were run on an Attune NxT flow cytometer (Thermo Fisher Scientific) and acquired data were analyzed using FlowJo software (FlowJo, LLC). Doublets were excluded on FSC‐A/FSC‐H dot plots. Live cells were defined as a negative for Zombie Yellow. Senescent vascular smooth muscle cells were identified as MYH11‐positive/Cell Event senescence‐positive cells in the histogram plot. Senescent T cells were identified as CD3‐positive cells/CellEvent senescence‐positive cells in the histogram plot. Senescent macrophage population was identified as F4/80‐positive cells/CellEvent senescence‐positive cells in the histogram plot. Senescent endothelial cells were identified as CD31‐positive cells/CellEvent TM senescence‐positive cells in the histogram plot.

### 4.9 Statistics

Unless otherwise noted, all values are reported as average ± SEM. Due to lack of normality, the one‐way non‐parametric Kruskal–Wallis test with Bonferroni correction for multiple comparisons was chosen to evaluate differences in geometrical, mechanical, and microstructural metrics across age groups. The non‐parametric Spearman's rank‐order correlation coefficient was used to further measure the strength and direction of association between increasing age and metrics of interest. This ensured that noted differences across age groups were not only statistically significant but also biologically meaningful. Significance difference in vasoconstriction parameters was evaluated using one‐way ANOVA. The effect of age, body mass, and their interaction on aortic geometry was assessed using one‐way ANCOVA with age group as the independent variable and body mass as the covariate. For scRNA‐seq data, *p* values were adjusted using the Benjamini‐Hochberg method for false discovery correction. Genes with an adjusted *p* < 0.05 were considered as differentially expressed.

## AUTHOR CONTRIBUTIONS

CB and BR conceptually developed and supervised all aspects of the project. CR and MS performed sequencing and analysis. CR performed flow cytometry, immunostaining, and histology. YF and JM performed inflation/extension and vasoactive testing. YF analyzed biomechanical data and quantified histology. YF, CB, and BR wrote the manuscript assisted by CR. YP, SM, and JV performed experiments and helped with graphical illustration.

## FUNDING INFORMATION

We acknowledge financial support from the National Institute of Health (R01HL146627 and R01HL149927 to BR and R01HL168719 and R21HL148747 to CB). CR is funded by American Heart Association pre‐doctoral fellowship (23PRE1023003). MS is funded by American Heart Association post‐doctoral fellowship (907602). YF is funded by NSF GRFP fellowship (1451070).

## CONFLICT OF INTEREST STATEMENT

No conflict to declare.

## Supporting information


Data S1:

## Data Availability

The authors declare that all data supporting the results in this study are reported within the manuscript and its supplementary information. Raw data will be either available from the corresponding authors on reasonable request or made publicly available on the GEO repository upon publication.
