# Supplementary material for: Mapping the unicellular transcriptome of the ascending thoracic aorta to changes in mechanosensing and mechanoadaptation during aging
Source: Aging Cell. 2024 Jun 2;23(8):e14197. doi: 10.1111/acel.14197 (PMC11320362; doi:10.1111/acel.14197)
Supplement: Supplementary file 1 — Data S1: [file ACEL-23-e14197-s001.pdf]

SUPPLEMENTARY MATERIAL

Mapping the unicellular transcriptome of the ascending thoracic aorta to changes in mechanosensing and mechanoadaptation during aging

Cristobal Rivera[2,3†], Yasmeen M. Farra[1†], Michele Silvestro[2,3], Steven Medvedovsky[2,3], Jacqueline Matz[1], John Vlahos[2,3], Bhama Ramkhelawon[2,3*], Chiara Bellini[1*]

[1]Department of Bioengineering, Northeastern University, Boston, MA, USA.

[2]Division of Vascular and Endovascular Surgery, Department of Surgery, New York University Langone Medical Center, New York, USA.

[3]Department of Cell Biology, New York University Langone Medical Center, New York, USA.

† These authors contributed equally to this work

* These authors contributed equally to this work

* Correspondence should be addressed to CB (c.bellini@northeastern.edu) and BR (bhama.ramkhelawon@nyulangone.org).

MATERIALS AND METHODS

Layer-specific tissue stiffness

Biaxial data were further analyzed using a bi-layered model of the aortic wall that features microstructurally-motivated and layer-specific constitutive relations and accounts for the deposition stretches of newly secreted constituents (e.g., elastic fibers or multiple families of collagen fibers) as they integrate within the existing matrix[1] in the homeostatic reference configuration. We assumed a mass-averaged strain energy function in the form:

$$W = \phi^e W^e(\mathbf{F}^e) + \phi^m W^m(\lambda^m) + \sum_{j=1}^{4} \phi^{c_j} W^{c_j}(\lambda^{c_j}), \qquad \text{(S1)}$$

where the superscripts $i = e, m$ and c refer to elastic fibers, smooth muscle bundles, and each of four families of collagen fibers ($j = 1,2,3,4$), respectively, ϕ^i are the mass fractions (from histological analysis, Figures 5 and S2) and W^i are the stored energy functions for the constituents that compose the mixture, $\mathbf{F}^e$ is the deformation gradient tensor experienced by the elastic fibers, and λ^m and λ^{c_j} are the stretches experienced by the smooth muscle and the jth family of collagen fibers, respectively. Similar to the bulk formulation in Equation 2, we described the elastic fiber behavior with a neo-Hookean stored energy function

$$W^e = \frac{c^e}{2}(I_{\mathbf{C}^e} - 3), \qquad \text{(S2)}$$

where c^e is a coefficient with the dimension of a stress, $I_{\mathbf{C}^e} = tr(\mathbf{C}^e)$, $\mathbf{C}^e = \mathbf{F}^{e\mathrm{T}}\mathbf{F}^e$ and $\mathbf{F}^e = \mathbf{F}\mathbf{G}_h^e$, with $\mathbf{F}$ the deformation gradient tensor that describes the deformation of the mixture as a whole and $\mathbf{G}_h^e$ the deposition stretch tensor between the natural (stress-free) configuration of the elastic fibers and the homeostatic reference configuration. $\mathbf{G}_h^e$ was assumed to be diagonal with circumferential and axial components within the [1.65, 1.74] range[2] and the radial stretch determined by enforcing incompressibility. The nonlinear responses of collagen fibers and circumferential smooth muscle were modeled as Fung exponentials,

$$W^m = \frac{c_1^m}{4c_2^m}\left[e^{c_2^m(IV^m-1)^2} - 1\right], \qquad \text{(S3)}$$

$$W^{c_j} = \frac{c_1^c}{4c_2^c}\left[e^{c_2^c\left(IV^{c_j}-1\right)^2} - 1\right], \qquad \text{(S4)}$$

where c_1^m and c_1^c are coefficients with the dimension of a stress, while c_2^m and c_2^c are dimensionless. Neither smooth muscle nor collagen fibers were assumed to have any radial component. The stretch experienced by smooth muscle was obtained by projecting $\boldsymbol{C}$ along the cell axis,

$$\lambda^{m} = \sqrt{IV^{m}} = \mathrm{G}_{h}^{m}\sqrt{\mathbf{C} : (\mathbf{M}^{m} \otimes \mathbf{M}^{m})}, \tag{S5}$$

where $\mathbf{M}^{m} = [0, \sin\alpha_0^{m}, \cos\alpha_0^{m}]$ is the reference smooth muscle orientation and G_{h}^{m} assumed values in the [1.03, 1.05] range[2]. Similarly, the stretch in the collagen fiber direction was determined as

$$\lambda^{c_j} = \sqrt{IV^{c_j}} = \mathrm{G}_{h}^{c_j}\sqrt{\mathbf{C} : (\mathbf{M}^{c_j} \otimes \mathbf{M}^{c_j})}, \tag{S6}$$

where $\mathbf{M}^{c_j} = [0, \sin\alpha_0^{c_j}, \cos\alpha_0^{c_j}]$ is the reference dominant orientation of the jth collagen fiber family and $\mathrm{G}_{h}^{c_j}$ varied within the [1.10, 1.13] range[2]. Also estimated from experimental data were two additional parameters for the proportion of circumferentially- (β_{ϑ}) and axially- (β_{z}) oriented collagen fibers. The 8 best-fit parameters were obtained via nonlinear regression on the average experimental data and used to predict the transmural distribution of linearized circumferential tissue stiffness following the small-on-large approach[3].

SUPPLEMENTARY TABLES

Table S1. Differentially expressed genes in cell clusters from single-cell RNA sequencing of C57BL/6J wildtype (WT) ascending thoracic aorta (ATA) tissues between 12 and 84 weeks of age.

	p_val	avg_log2FC	pct.1	pct.2	p_val_adj	cluster	gene
1	0	2.11258627	0.952	0.204	0	T cells 1	Satb1
2	0	2.02736944	0.764	0.147	0	T cells 1	Rag1
3	0	1.93321659	0.923	0.200	0	T cells 1	Ptprc
4	0	1.71568400	0.786	0.141	0	T cells 1	Gm4258
5	0	1.64489619	0.649	0.144	0	T cells 1	Trbc1
6	0	1.63747668	0.748	0.176	0	T cells 1	Trbc2
7	0	1.58931438	0.844	0.154	0	T cells 1	Lck
8	0	1.51048926	0.735	0.102	0	T cells 1	Ccr9
9	0	1.46889296	0.764	0.158	0	T cells 1	Arpp21
10	0	1.44890257	0.687	0.134	0	T cells 1	Cd8b1
11	0	1.44607587	0.801	0.167	0	T cells 1	Tcf7
12	0	1.37560119	0.741	0.135	0	T cells 1	Themis
13	0	1.25030507	0.687	0.154	0	T cells 1	Trp53inp1
14	0	1.23369026	0.693	0.156	0	T cells 1	Glcci1
15	0	1.19397030	0.683	0.182	0	T cells 1	Rmnd5a
16	0	1.18302711	0.767	0.272	0	T cells 1	Ets1
17	0	1.16656162	0.669	0.120	0	T cells 1	Cd8a
18	0	1.12541670	0.683	0.143	0	T cells 1	Bcl11b
19	0	1.07944274	0.696	0.286	0	T cells 1	Mier1
20	0	1.03753480	0.673	0.168	0	T cells 1	Thy1
21	0	1.70766648	1.000	0.839	0	VSMC 1	Mgp
22	0	1.45586254	0.947	0.350	0	VSMC 1	Pam
23	0	1.42896402	1.000	0.867	0	VSMC 1	Acta2
24	0	1.38361597	0.994	0.643	0	VSMC 1	Tpm2
25	0	1.36612968	0.999	0.739	0	VSMC 1	Tagln
26	0	1.27778162	0.999	0.976	0	VSMC 1	Actb
27	0	1.27675922	0.976	0.526	0	VSMC 1	Csrp2
28	0	1.27032018	1.000	0.937	0	VSMC 1	Myl6
29	0	1.25789565	0.988	0.613	0	VSMC 1	Myh11
30	0	1.25544313	0.975	0.557	0	VSMC 1	Myl9
31	0	1.24268346	1.000	0.963	0	VSMC 1	Fth1
32	0	1.23184533	0.996	0.728	0	VSMC 1	Vim
33	0	1.20257427	0.978	0.613	0	VSMC 1	Cald1
34	0	1.17563010	0.985	0.646	0	VSMC 1	Dstn
35	0	1.13269719	0.961	0.524	0	VSMC 1	Bgn

36	0	1.13081374	0.978	0.614	0	VSMC 1	S100a6
37	0	1.11279959	0.970	0.568	0	VSMC 1	Tpm1
38	0	1.10819911	0.955	0.611	0	VSMC 1	Lgals1
39	0	1.07363163	0.985	0.645	0	VSMC 1	Igfbp7
40	0	1.03118651	0.861	0.316	0	VSMC 1	Nupr1
41	0	1.67448606	0.986	0.745	0	VSMC 2	Tagln
42	0	1.54918803	0.994	0.870	0	VSMC 2	Acta2
43	0	1.45731882	0.973	0.621	0	VSMC 2	Myh11
44	0	1.34038121	0.900	0.326	0	VSMC 2	Itga8
45	0	1.31684029	0.963	0.574	0	VSMC 2	Tpm1
46	0	1.29247376	0.904	0.364	0	VSMC 2	Lpp
47	0	1.26514746	0.937	0.539	0	VSMC 2	Csrp2
48	0	1.25640690	0.819	0.335	0	VSMC 2	Ccn2
49	0	1.22093084	0.844	0.323	0	VSMC 2	Eln
50	0	1.21657807	0.973	0.618	0	VSMC 2	Cald1
51	0	1.20653607	0.857	0.235	0	VSMC 2	Pcdh7
52	0	1.18509027	0.911	0.358	0	VSMC 2	Mylk
53	0	1.17062139	0.943	0.568	0	VSMC 2	Myl9
54	0	1.17052206	0.874	0.299	0	VSMC 2	Map1b
55	0	1.15134649	0.954	0.509	0	VSMC 2	Flna
56	0	1.13506858	0.995	0.964	0	VSMC 2	Fth1
57	0	1.13064447	0.895	0.349	0	VSMC 2	Fblim1
58	0	1.12956193	0.821	0.252	0	VSMC 2	Npnt
59	0	1.12792110	0.859	0.347	0	VSMC 2	Cnn1
60	0	1.12643491	0.961	0.654	0	VSMC 2	Tpm2
61	0	4.65519245	1.000	0.458	0	EC	Fabp4
62	0	2.86579244	0.939	0.080	0	EC	Cd36
63	0	2.62838497	0.914	0.052	0	EC	Gpihbp1
64	0	2.09467818	0.675	0.106	0	EC	Aqp1
65	0	2.02128687	0.855	0.074	0	EC	Ly6c1
66	0	2.00927789	0.838	0.057	0	EC	Flt1
67	0	1.98758855	0.761	0.040	0	EC	Cldn5
68	0	1.93955818	0.838	0.093	0	EC	Cavin2
69	0	1.88525785	0.814	0.117	0	EC	Klf2
70	0	1.84553708	0.793	0.030	0	EC	Kdr
71	0	1.81839530	0.827	0.042	0	EC	Cdh5
72	0	1.80804925	0.840	0.119	0	EC	Gng11
73	0	1.79072525	0.929	0.387	0	EC	Sptbn1
74	0	1.76484448	0.789	0.070	0	EC	Slfn5
75	0	1.75537157	0.780	0.054	0	EC	Ptprb

76	0	1.75298271	0.816	0.142	0	EC	Rgcc
77	0	1.74601405	0.895	0.270	0	EC	Cav1
78	0	1.71983147	0.721	0.106	0	EC	Cxcl12
79	0	1.68152454	0.802	0.054	0	EC	Egfl7
80	0	1.67563719	0.792	0.039	0	EC	Adgrf5
81	0	3.40843853	0.845	0.123	0	T cells 2	Hist1h2ap
82	0	3.28020421	0.947	0.192	0	T cells 2	Hmgb2
83	0	2.79977732	0.893	0.035	0	T cells 2	Mki67
84	0	2.78634403	0.853	0.033	0	T cells 2	Top2a
85	0	2.76897573	0.736	0.088	0	T cells 2	Hist1h1b
86	0	2.58715867	0.641	0.063	0	T cells 2	Hist1h2ae
87	0	2.14162231	0.876	0.112	0	T cells 2	Stmn1
88	0	2.13035366	0.994	0.865	0	T cells 2	Ptma
89	0	1.88643397	0.713	0.025	0	T cells 2	Cenpf
90	0	1.79277352	0.795	0.080	0	T cells 2	Hmgn2
91	0	1.78907380	0.868	0.286	0	T cells 2	H2afz
92	0	1.71818325	0.707	0.017	0	T cells 2	Pclaf
93	0	1.61628638	0.960	0.704	0	T cells 2	Hmgb1
94	0	1.61241547	0.559	0.030	0	T cells 2	Ube2c
95	0	1.59527230	0.660	0.180	0	T cells 2	Hist1h1e
96	0	1.55438232	0.695	0.014	0	T cells 2	Knl1
97	0	1.49813740	0.573	0.012	0	T cells 2	Hist1h3c
98	0	1.43126751	0.828	0.185	0	T cells 2	Anp32e
99	0	1.42344474	0.831	0.254	0	T cells 2	Tubb5
100	0	1.38329707	0.812	0.242	0	T cells 2	Nucks1
101	0	5.42979212	1.000	0.785	0	RBC	Hbb-bs
102	0	5.26355461	1.000	0.491	0	RBC	Hba-a1
103	0	5.17717777	1.000	0.425	0	RBC	Hba-a2
104	0	5.09086508	0.991	0.403	0	RBC	Hbb-bt
105	0	2.17507784	0.840	0.062	0	RBC	Alas2
106	0	1.69887322	0.654	0.072	0	RBC	Bpgm
107	0	1.69235986	0.671	0.115	0	RBC	Mkrn1
108	0	1.60526525	0.697	0.038	0	RBC	Snca
109	0	1.31533853	0.648	0.055	0	RBC	Tent5c
110	0	1.20315421	0.916	0.547	0	RBC	Ftl1
111	0	1.02078200	0.666	0.235	0	RBC	Bnip3l
112	0	1.01155251	0.568	0.035	0	RBC	Ube2l6
113	0	0.90308400	0.640	0.215	0	RBC	Epb41
114	0	0.85102337	0.553	0.127	0	RBC	Slc25a37
115	0	0.80801509	0.448	0.028	0	RBC	Isg20

116	0	0.70643238	0.460	0.058	0	RBC	Fech
117	4.20E-234	0.78282499	0.608	0.244	7.98E-230	RBC	Gabarapl2
118	2.70E-190	0.96155684	0.750	0.469	5.14E-186	RBC	Sec61g
119	6.58E-162	0.74847832	0.825	0.604	1.25E-157	RBC	Oaz1
120	1.81E-52	1.10096941	0.543	0.415	3.43E-48	RBC	Gpx1
121	0	4.18230135	0.982	0.269	0	Fibroblasts	Dcn
122	0	3.67094178	0.979	0.421	0	Fibroblasts	Gsn
123	0	2.73387177	0.771	0.092	0	Fibroblasts	Clec3b
124	0	2.42608339	0.665	0.068	0	Fibroblasts	Pi16
125	0	2.35519159	0.857	0.076	0	Fibroblasts	Dpt
126	0	2.34184569	0.652	0.100	0	Fibroblasts	C3
127	0	2.33810977	0.827	0.077	0	Fibroblasts	Lum
128	0	2.22731019	0.901	0.158	0	Fibroblasts	Serping1
129	0	2.18946507	0.898	0.167	0	Fibroblasts	Igfbp4
130	0	2.01918654	0.827	0.177	0	Fibroblasts	Mfap5
131	0	1.95724198	0.818	0.053	0	Fibroblasts	Serpinf1
132	0	1.92195725	0.940	0.353	0	Fibroblasts	Col1a2
133	0	1.84796165	0.946	0.334	0	Fibroblasts	Timp2
134	0	1.77630576	0.770	0.120	0	Fibroblasts	Col14a1
135	0	1.67306129	0.854	0.248	0	Fibroblasts	Col1a1
136	0	1.66611782	0.736	0.053	0	Fibroblasts	Fbln1
137	2.85E-292	1.94009916	0.916	0.404	5.42E-288	Fibroblasts	Col3a1
138	1.87E-239	2.40286599	0.561	0.145	3.56E-235	Fibroblasts	Igfbp5
139	3.28E-166	1.74820363	0.741	0.342	6.24E-162	Fibroblasts	Apoe
140	5.97E-106	1.87801122	0.960	0.796	1.13E-101	Fibroblasts	Cst3
141	0	4.08448974	0.858	0.063	0	Macrophages	Cd74
142	0	2.99841765	0.739	0.030	0	Macrophages	H2-Aa
143	0	2.92579173	0.737	0.030	0	Macrophages	H2-Ab1
144	0	2.77732929	0.695	0.028	0	Macrophages	H2-Eb1
145	0	2.67601646	0.551	0.038	0	Macrophages	Lyz2
146	0	2.32596583	0.459	0.018	0	Macrophages	C1qa
147	0	1.94014670	0.405	0.016	0	Macrophages	C1qb
148	0	1.72780020	0.404	0.012	0	Macrophages	C1qc
149	0	1.66585805	0.358	0.024	0	Macrophages	Pf4
150	0	1.54424941	0.425	0.009	0	Macrophages	Mrc1
151	0	1.43126345	0.430	0.012	0	Macrophages	Fcer1g
152	0	1.30480183	0.410	0.012	0	Macrophages	Tyrobp
153	0	1.17447092	0.454	0.006	0	Macrophages	Ctss
154	2.26E-94	3.63662585	0.221	0.043	4.30E-90	Macrophages	Igkc
155	1.58E-91	1.15740923	0.434	0.153	3.01E-87	Macrophages	Cd52

156	1.04E-76	2.26460856	0.616	0.348	1.98E-72	Macrophages	Apoe
157	2.75E-56	1.17490893	0.524	0.311	5.23E-52	Macrophages	Ctsb
158	8.59E-54	2.46460327	0.392	0.171	1.63E-49	Macrophages	Ighm
159	4.04E-52	1.13666527	0.750	0.565	7.69E-48	Macrophages	Ftl1
160	2.13E-39	1.24474355	0.452	0.282	4.06E-35	Macrophages	Selenop
161	0	3.69287716	0.912	0.057	0	Platelets	Cytl1
162	0	2.03675929	0.846	0.013	0	Platelets	Vwf
163	0	1.77062553	0.588	0.042	0	Platelets	Sfrp1
164	0	1.75899629	0.614	0.017	0	Platelets	Bmp4
165	0	1.73855497	0.711	0.051	0	Platelets	Vcam1
166	0	1.62978394	0.789	0.044	0	Platelets	Cgnl1
167	0	1.53676474	0.763	0.043	0	Platelets	Lmo2
168	2.11E-287	2.07113894	0.939	0.136	4.01E-283	Platelets	Ptprb
169	1.70E-285	1.53882998	0.851	0.116	3.24E-281	Platelets	Ece1
170	1.15E-270	2.45798647	0.978	0.184	2.19E-266	Platelets	Pecam1
171	9.97E-263	1.50545695	0.864	0.127	1.90E-258	Platelets	Heg1
172	1.29E-258	1.62835880	0.860	0.125	2.45E-254	Platelets	Ramp2
173	2.76E-218	1.38804852	0.746	0.113	5.24E-214	Platelets	Ctsh
174	2.05E-169	2.44877788	0.952	0.324	3.90E-165	Platelets	Cfh
175	6.12E-137	1.42986809	0.654	0.123	1.16E-132	Platelets	Ctla2a
176	1.60E-126	2.90440791	0.912	0.350	3.03E-122	Platelets	Apoe
177	1.65E-125	1.96702631	0.877	0.321	3.14E-121	Platelets	Clu
178	2.18E-122	2.35338305	0.969	0.481	4.14E-118	Platelets	Tm4sf1
179	3.53E-120	1.62110637	0.904	0.302	6.71E-116	Platelets	H2-K1
180	4.28E-113	1.48974836	0.930	0.345	8.13E-109	Platelets	H2-D1
181	0	3.85275356	0.907	0.046	0	Myofibroblasts	Igfbp6
182	0	2.90733443	0.898	0.006	0	Myofibroblasts	Upk3b
183	0	2.35531153	0.831	0.013	0	Myofibroblasts	Gpm6a
184	0	2.13138546	0.788	0.004	0	Myofibroblasts	Msln
185	0	2.08562954	0.737	0.004	0	Myofibroblasts	Muc16
186	0	1.94992684	0.797	0.047	0	Myofibroblasts	Efemp1
187	0	1.85180687	0.559	0.009	0	Myofibroblasts	Myl7
188	0	1.78438086	0.653	0.005	0	Myofibroblasts	Nkain4
189	0	1.60349233	0.653	0.012	0	Myofibroblasts	Hpgd
190	1.90E-242	1.63856145	0.712	0.048	3.62E-238	Myofibroblasts	Gpc3
191	8.58E-238	1.83139660	0.797	0.063	1.63E-233	Myofibroblasts	Gas1
192	3.98E-210	3.23055056	0.966	0.115	7.57E-206	Myofibroblasts	C3
193	8.48E-182	4.63311164	0.992	0.155	1.61E-177	Myofibroblasts	Igfbp5
194	2.53E-147	1.88659160	0.771	0.099	4.80E-143	Myofibroblasts	Rnase4
195	5.98E-116	1.93206949	0.856	0.166	1.14E-111	Myofibroblasts	Sulf1

196	1.32E-83	2.98575953	0.932	0.332	2.52E-79	Myofibroblasts	Rarres2
197	4.79E-76	1.91707508	0.966	0.361	9.11E-72	Myofibroblasts	Aebp1
198	8.48E-76	1.79767831	0.941	0.291	1.61E-71	Myofibroblasts	Dcn
199	1.09E-62	2.44506682	0.983	0.707	2.07E-58	Myofibroblasts	Crip1
200	1.34E-61	1.70228219	0.831	0.285	2.55E-57	Myofibroblasts	Bsg
201	0	2.84845464	0.833	0.028	0	Adipocytes	Cidea
202	0	2.63153696	0.939	0.021	0	Adipocytes	Pck1
203	3.65E-270	3.97722983	0.909	0.042	6.94E-266	Adipocytes	Cfd
204	1.70E-224	2.59773419	0.833	0.042	3.23E-220	Adipocytes	Fasn
205	1.62E-202	2.55201430	0.773	0.040	3.09E-198	Adipocytes	Ucp1
206	6.22E-199	3.35619519	0.939	0.063	1.18E-194	Adipocytes	Car3
207	5.58E-185	3.32087828	0.879	0.058	1.06E-180	Adipocytes	Cox8b
208	2.74E-178	2.42953851	0.636	0.030	5.21E-174	Adipocytes	Cox7a1
209	2.51E-141	3.89819613	0.788	0.062	4.77E-137	Adipocytes	Scd1
210	4.02E-71	2.33600176	0.742	0.105	7.64E-67	Adipocytes	Chchd10
211	2.17E-44	3.22170769	1.000	1.000	4.13E-40	Adipocytes	mt-Co1
212	3.31E-43	2.98876531	1.000	1.000	6.30E-39	Adipocytes	mt-Co3
213	1.57E-42	3.04492046	1.000	1.000	2.99E-38	Adipocytes	mt-Co2
214	4.27E-42	2.60977993	1.000	1.000	8.12E-38	Adipocytes	mt-Nd1
215	5.35E-42	2.98513526	1.000	1.000	1.02E-37	Adipocytes	mt-Nd4
216	1.60E-41	3.20078205	1.000	1.000	3.04E-37	Adipocytes	mt-Atp6
217	1.75E-41	3.13601181	1.000	1.000	3.33E-37	Adipocytes	mt-Cytb
218	2.13E-41	3.01688300	1.000	1.000	4.05E-37	Adipocytes	mt-Nd2
219	1.37E-37	2.26363407	1.000	0.998	2.61E-33	Adipocytes	mt-Nd4l
220	3.07E-34	2.75034454	0.621	0.143	5.83E-30	Adipocytes	Ifi27l2a
221	0	5.38757391	0.958	0.013	0	Undefined	Wfdc2
222	0	5.18149373	1.000	0.009	0	Undefined	Cyp2f2
223	0	4.58337493	0.667	0.007	0	Undefined	Reg3g
224	0	4.44498247	0.479	0.004	0	Undefined	Ltf
225	0	3.52427251	0.917	0.006	0	Undefined	Lypd2
226	0	3.40500776	0.521	0.003	0	Undefined	Bpifb1
227	0	3.32840050	0.917	0.021	0	Undefined	Gsto1
228	0	2.60630159	0.417	0.002	0	Undefined	Sftpa1
229	0	2.55079923	0.583	0.002	0	Undefined	Cyp2a5
230	0	2.42946682	0.854	0.001	0	Undefined	Sult1d1
231	6.73E-236	3.20113892	0.854	0.031	1.28E-231	Undefined	Aldh1a1
232	1.37E-153	6.47607610	0.771	0.039	2.60E-149	Undefined	Scgb3a2
233	1.31E-122	2.60916717	0.875	0.066	2.50E-118	Undefined	Tmem176a
234	5.40E-113	6.08623687	0.625	0.035	1.03E-108	Undefined	Scgb3a1
235	2.03E-95	2.32833229	0.875	0.085	3.86E-91	Undefined	Tmem176b

236	8.36E-89	8.27235156	0.771	0.071	1.59E-84	Undefined	Bpifa1
237	1.13E-82	3.82577618	0.979	0.141	2.14E-78	Undefined	Cbr2
238	3.22E-73	2.47860888	0.896	0.127	6.11E-69	Undefined	Prdx6
239	3.80E-42	3.14493293	0.479	0.052	7.21E-38	Undefined	Lyz2
240	1.48E-37	8.15782079	0.854	0.219	2.81E-33	Undefined	Scgb1a1

Table S2. Average (± SEM) age, body mass, and blood pressure across genotypes and age groups.

	Body Mass (g)	Blood Pressure (mmHg)		Age		
		Systolic	Diastolic	Days	Weeks	Months
**WT**						
12 weeks	19.9 ± 0.4	113 ± 6	81 ± 6	86 ± 1	12.3 ± 0.2	2.83 ± 0.05
26 weeks	23.2 ± 0.8	118 ± 5	88 ± 2	179 ± 2	25.5 ± 0.3	5.87 ± 0.07
49 weeks	26.5 ± 1.0	112 ± 3	76 ± 3	343 ± 4	48.9 ± 0.5	11.25 ± 0.12
68 weeks	30.7 ± 1.3	106 ± 1	76 ± 2	476 ± 1	68.0 ± 0.2	15.65 ± 0.05
84 weeks	30.0 ± 0.7	107 ± 3	70 ± 7	590 ± 6	84.3 ± 0.8	19.40 ± 0.18
**VSMC$^{\Delta Piezo1}$**						
12 weeks	20.7 ± 0.8	99 ± 6	70 ± 4	89 ± 1	12.7 ± 0.1	2.93 ± 0.01
100 weeks	27.5 ± 0.7	97 ± 2	61 ± 1	706 ± 3	100.8 ± 0.5	23.18 ± 0.11

Table S3. Best-fit coefficients of the four-fiber family strain energy potential (Equation 2) as estimated from biaxial experimental data to describe the bulk mechanical response of the ATA at the 12-, 26-, 49-, 68-, and 84-week endpoints in WT mice and at the 12- and 100-week endpoints in VSMC$^{\Delta Piezo\text{-}1}$ mice.

	Elastic Fibers	Axial Collagen		Circumferential Collagen + SMC		Symmetric Diagonal Collagen			Error
	$c\ (kPa)$	$c_1^1\ (kPa)$	c_2^1	$c_1^2\ (kPa)$	c_2^2	$c_1^{3,4}\ (kPa)$	$c_2^{3,4}$	α_0 (deg)	$RMSE$
**WT**									
12 weeks	19.151	13.540	2.3E-14	16.198	8.2E-13	14.442	0.374	46.043	0.052
26 weeks	26.647	8.299	2.3E-14	18.411	0.068	10.848	0.392	47.014	0.051
49 weeks	8.845	13.792	5.3E-04	4.6E-08	6.721	18.439	0.291	52.677	0.061
68 weeks	6.319	17.764	2.8E-11	1.0E-04	5.005	26.953	0.359	51.451	0.065
84 weeks	7.106	18.614	0.017	3.1E-05	6.526	23.878	0.538	51.589	0.051
**VSMC$^{\Delta Piezo\text{-}1}$**									
12 weeks	28.257	5.190	0.185	2.3E-11	4.624	12.695	0.265	48.373	0.069
100 weeks	17.574	8.765	0.003	1.5E-08	4.869	13.730	0.350	50.292	0.062

Table S4. Best-fit parameters of a mass-averaged form of the four-fiber family strain energy potential (Equations S1-4) as estimated from biaxial experimental data to describe the layer-specific mechanical response of the WT ATA at the 12- and 84- week endpoints.

	Elastic Fibers	SMCs		Collagen Fibers					Error
	$c^e\ (kPa)$	$c_1^m\ (kPa)$	c_2^m	$c_1^c\ (kPa)$	c_2^c	α_0 (deg)	β_ϑ	β_z	$RMSE$
**WT**									
12 weeks	179.660	471.226	4.711	4389.129	1.232	45.948	0.072	0.031	0.052
84 weeks	266.285	497.360	60.069	1279.611	8.379	46.971	0.011	0.014	0.054

Table S5. Morphological and mechanical properties of the ATA at the 12-, 26-, 49-, 68-, and 84-week endpoints in WT mice. Statistical significance for the difference between age groups is denoted by * for $p < 0.05$ *vs.* 12 weeks, † for $p < 0.05$ *vs.* 26 weeks, ‡ for $p < 0.05$ *vs.* 49 weeks, and § for $p < 0.05$ *vs.* 68 weeks. Spearman correlation coefficients (r_s) that relate mechanical metrics to age are also reported, with significance indicated by ¥ for $p < 0.05$.

	WT					
	12 weeks	*26 weeks*	*49 weeks*	*68 weeks*	*84 weeks*	Spearman
n	6	8	7	8	14	r_s
Distensibility (MPa^{-1})	25.0 ± 1.8	24.7 ± 1.6	15.4 ± 0.5	15.8 ± 1.0	10.6 ± 0.8 *†	-0.859 ¥
Traction-free configuration						
Geometry						
Outer Diameter (µm)	1115 ± 26	1109 ± 32	1186 ± 37	1176 ± 27	1353 ± 25 *†§	0.752 ¥
Wall Thickness (µm)	111 ± 1	108 ± 6	128 ± 2	128 ± 2	146 ± 5 *†	0.808 ¥
Loaded systolic configuration						
Geometry						
Outer Diameter (µm)	1639 ± 24	1642 ± 15	1737 ± 42	1646 ± 64	1912 ± 23 *†§	0.688 ¥
Wall Thickness (µm)	40 ± 1	40 ± 4	51 ± 3	54 ± 1	65 ± 2 *†	0.759 ¥
Stretch (-)						
Circumferential	1.60 ± 0.03	1.61 ± 0.05	1.62 ± 0.04	1.57 ± 0.03	1.53 ± 0.02 †	-0.275
Axial	1.76 ± 0.04	1.72 ± 0.04	1.68 ± 0.05	1.51 ± 0.02 †	1.47 ± 0.03 *†‡	-0.738 ¥
Cauchy Stress (kPa)						
Circumferential	315 ± 11	331 ± 27	261 ± 15	160 ± 7 †	222 ± 7 †	-0.574 ¥
Axial	316 ± 14	304 ± 23	242 ± 11	188 ± 13 †	177 ± 9 *†	-0.707 ¥
Linearized Stiffness (MPa)						
Circumferential	1.98 ± 0.14	2.24 ± 0.23	2.66 ± 0.21	2.55 ± 0.42	3.73 ± 0.40 *	0.521 ¥
Axial	1.78 ± 0.11	1.74 ± 0.13	1.42 ± 0.06	1.33 ± 0.11	1.10 ± 0.06 *†	-0.695 ¥
Stored Energy (kPa)	88 ± 3	90 ± 10	61 ± 4	48 ± 4 †	38 ± 2 *†‡	-0.792 ¥

Table S6. Morphological and mechanical properties of ATA at the 12- and 100-week endpoints in VSMC$^{\Delta Piezo\text{-}1}$ mice. Statistical significance for the difference between age groups is denoted by * for $p < 0.05$ *vs.* 12 weeks.

	**vSMC$^{\Delta Piezo\text{-}1}$**		
	12 weeks	*100 weeks*	
n	5	7	
Distensibility (MPa^{-1})	26.3 ± 0.7	20.0 ± 0.7	*
Traction-free configuration			
Geometry			
Outer Diameter (µm)	1053 ± 28	1196 ± 24	*
Wall Thickness (µm)	115 ± 2	122 ± 3	
Loaded systolic configuration			
Geometry			
Outer Diameter (µm)	1674 ± 37	1828 ± 35	*
Wall Thickness (µm)	39 ± 2	43 ± 1	
Stretch (-)			
Circumferential	1.75 ± 0.02	1.66 ± 0.03	*
Axial	1.70 ± 0.04	1.73 ± 0.05	
Cauchy Stress (kPa)			
Circumferential	329 ± 17	329 ± 9	
Axial	306 ± 24	320 ± 33	
Linearized Stiffness (MPa)			
Circumferential	2.07 ± 0.16	2.48 ± 0.11	
Axial	1.64 ± 0.17	1.76 ± 0.24	
Stored Energy (kPa)	92 ± 7	81 ± 5	

SUPPLEMENTARY FIGURES

Figure S1

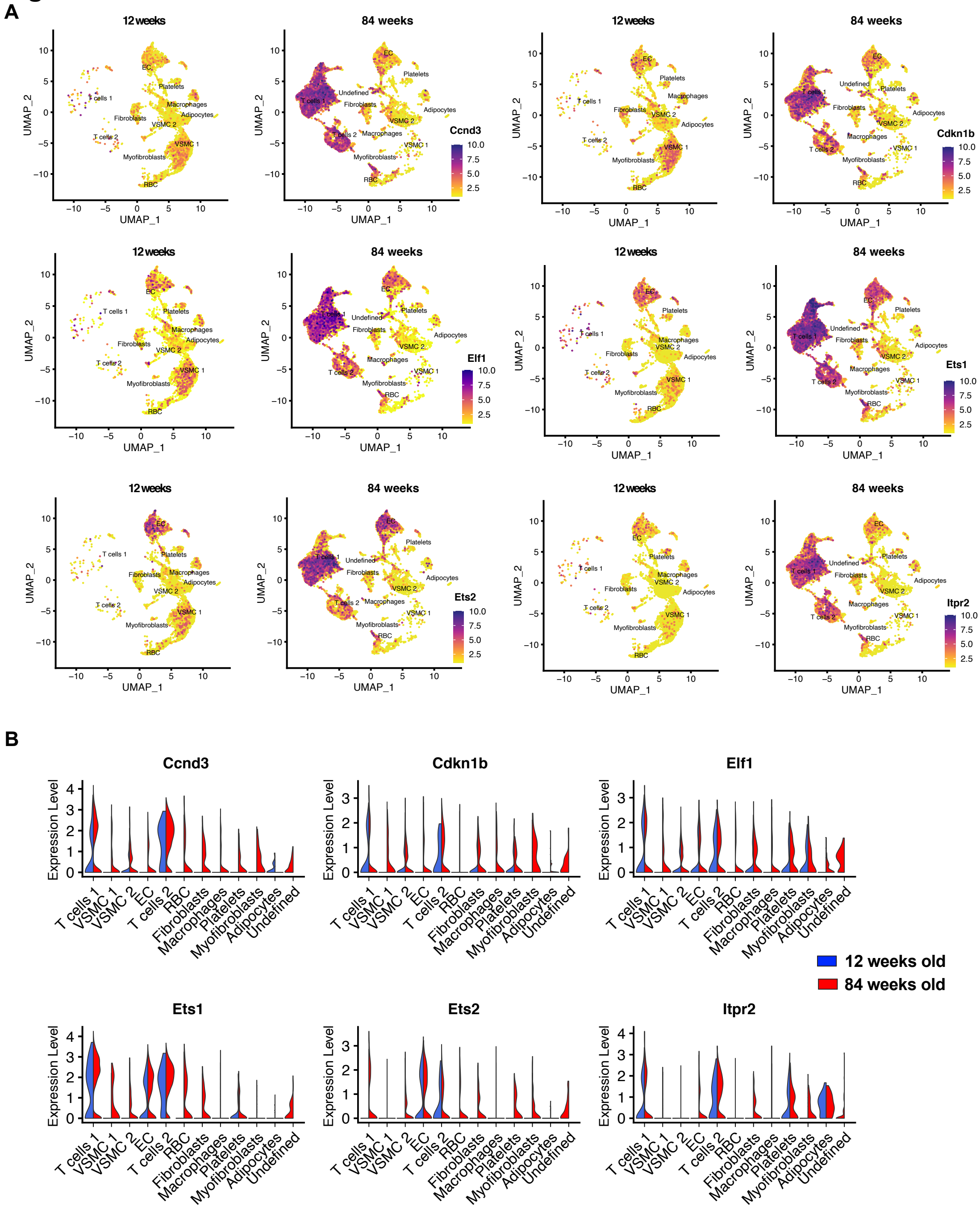

Figure S1. Senescence markers per cluster. (A) UMAP visualization showing expression of senescence markers (*Ccnd3*, *Cdkn1b*, *Elf1*, *Ets1*, *Ets2*, *Itpr2*) and violin plots (B) in different cell clusters in ATA tissues from WT mice aged 12 and 84 weeks. ATA samples were obtained from 12- (n = 3) and 84- (n = 3) week-old mice for single-cell RNA sequencing.

Figure S2

A

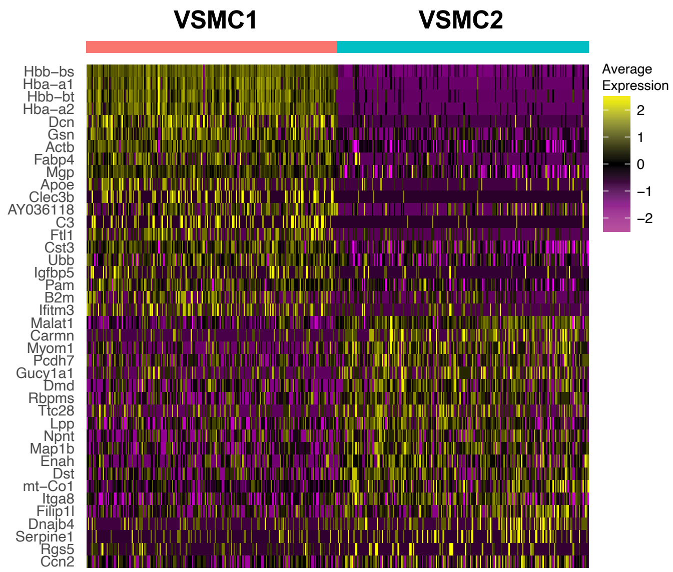

Figure S2. Heatmap displaying the top 20 differentially expressed transcripts for each sub-cluster of VSMC (VSMC1 and 2). ATA samples were obtained from 12- (n = 3) and 84- (n = 3) week-old WT mice for single-cell RNA sequencing.

Figure S3

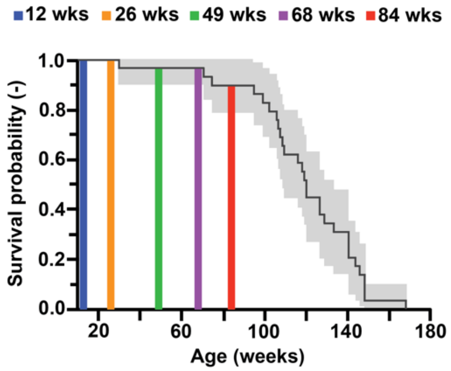

Figure S3. Survival curve for female WT mice, recreated with data from the Jackson Laboratory Yuan2 dataset (RRID: SCR_003212). The grey shaded area envelopes the 95% confidence interval. The endpoints for this study are visualized by vertical bars and cover most of the mouse adult lifespan, before the probability of survival begins to decline after ~100 weeks of age.

Figure S4

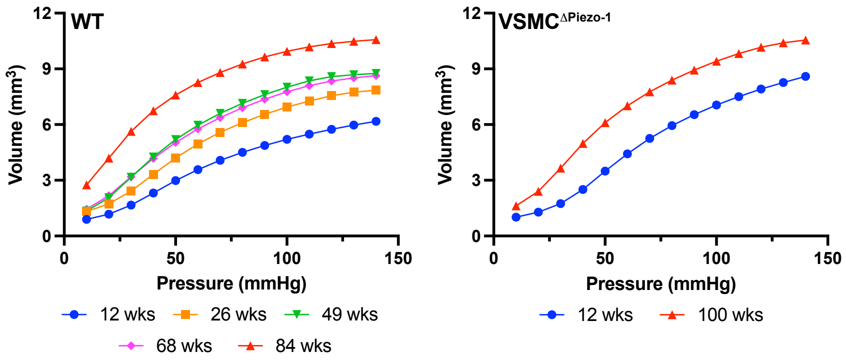

Figure S4. Predicted luminal volume vs. pressure behavior for the representative WT and VSMC$^{Piezo\text{-}1}$ ATA at selected endpoints. Compliance curves progressively shift upward with aging.

Figure S5

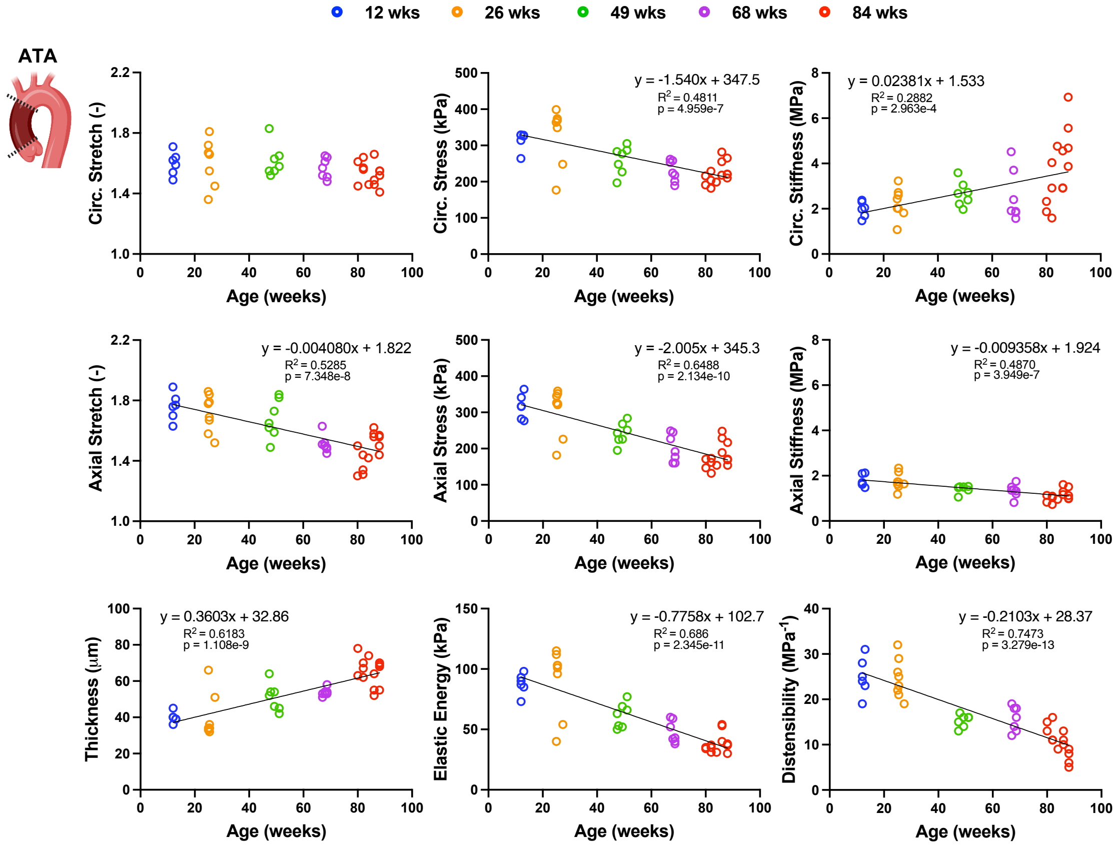

Figure S5. Age-dependent variation in the material, geometrical, and structural properties of WT ATA samples. Metrics of stretch, stress, stiffness, thickness, and energy are calculated at group-specific values of systolic pressure. Cyclic distensibility accounts for group-specific values of luminal pressure and diameter between diastole and systole. Circumferential stiffness and thickness increase with advancing age, while circumferential stress, axial stretch, axial stress, axial stiffness, elastic energy, and cyclic distensibility exhibit a negative correlation with age. Circumferential stretch is preserved with age. Group sizes for the passive mechanical analysis are: 12 weeks (n = 6), 26 weeks (n = 8), 49 weeks (n = 8), 68 weeks (n = 9), and 84 weeks (n = 19). Linear regression lines visualize trends on age-dependent metrics.

Figure S6

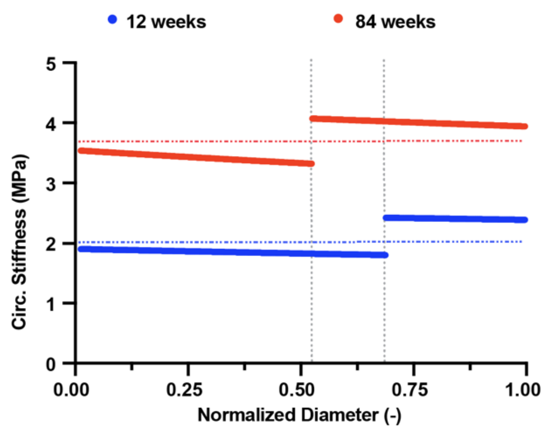

Figure S6. Predicted linearized circumferential stiffness of tissues in the medial and adventitial layers of the representative 12- and 84-week-old WT ATA. Aging is associated with circumferential tissue stiffening in both the media and adventitial layers of the wall. Note, horizontal dash-dotted lines indicate through-thickness integral mean values of circumferential stiffness for the two groups, closely matching the predictions of the transmurally-averaged model (Table S5). Grey vertical dotted lines define the medial/adventitial border in the two groups.

Figure S7

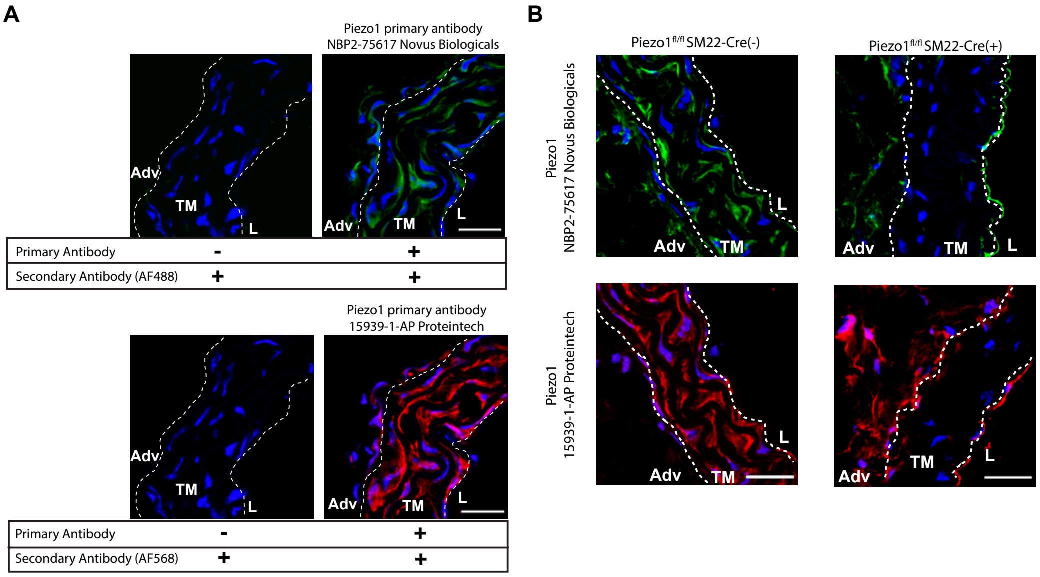

Figure S7. Validation anti-Piezo-1 antibodies. Representative images of Piezo-1 staining in aged ATA sections of WT and VSMC-null Piezo-1 mice in the presence or not of antibodies, as indicated. n = 5/sample/genotype.

Figure S8

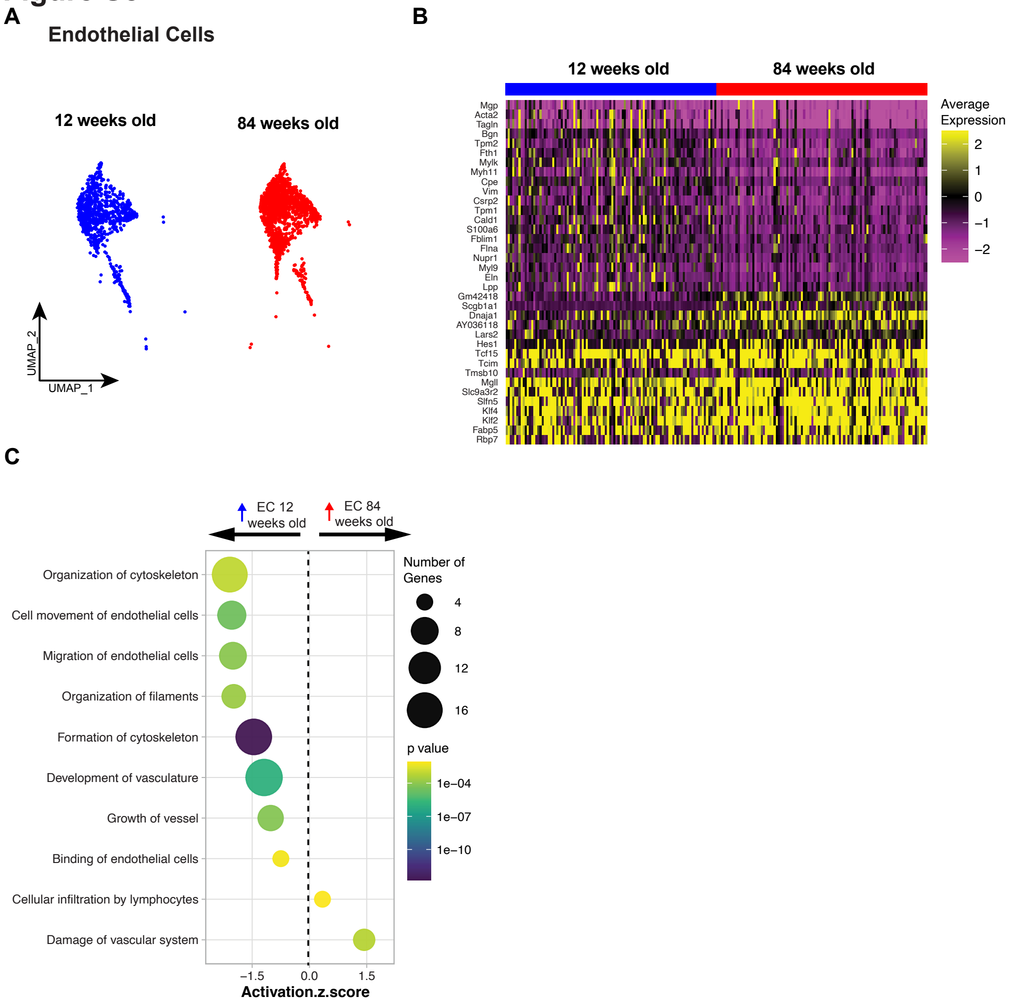

Figure S8. (A) UMAP of Endothelial Cells (EC) clusters in the WT ATA of 12- and 84-week-old mice. (B) Heatmap displaying the top 20 differentially expressed transcripts for EC per age group. (C) Upregulated signaling pathways of EC cluster in ATA samples between ages as indicated. n = 3/age group.

REFERENCES

1. Bellini, C., Ferruzzi, J., Roccabianca, S., Di Martino, E. S. & Humphrey, J. D. A microstructurally motivated model of arterial wall mechanics with mechanobiological implications. *Ann. Biomed. Eng.* **42**, 488–502 (2014).
2. Bersi, M. R. *et al.* Excessive Adventitial Remodeling Leads to Early Aortic Maladaptation in Angiotensin-Induced Hypertension. *Hypertension* **67**, 890–896 (2016).
3. Baek, S., Gleason, R. L., Rajagopal, K. R. & Humphrey, J. D. Theory of small on large: Potential utility in computations of fluid–solid interactions in arteries. *Comput. Methods Appl. Mech. Eng.* **196**, 3070–3078 (2007).
